# Covalent Bonding of Pyrrolobenzodiazepines (PBDs) to Terminal Guanine Residues within Duplex and Hairpin DNA Fragments

**DOI:** 10.1371/journal.pone.0152303

**Published:** 2016-04-07

**Authors:** Julia Mantaj, Paul J. M. Jackson, Kersti Karu, Khondaker M. Rahman, David E. Thurston

**Affiliations:** 1 Institute of Pharmaceutical Science, King’s College London, 7 Trinity Street, London, SE1 1DB, United Kingdom; 2 Femtogenix Limited, Britannia House, 7 Trinity Street, London, SE1 1DB, United Kingdom; 3 UCL Chemistry Mass Spectrometry Facility, Christopher Ingold Building, Chemistry Department, 20 Gordon Street, London, WC1H 0AJ, United Kingdom; Lawrence Berkeley National Lab, UNITED STATES

## Abstract

Pyrrolobenzodiazepines (PBDs) are covalent-binding DNA-interactive agents with growing importance as payloads in Antibody Drug Conjugates (ADCs). Until now, PBDs were thought to covalently bond to C2-NH_2_ groups of guanines in the DNA-minor groove across a three-base-pair recognition sequence. Using HPLC/MS methodology with designed hairpin and duplex oligonucleotides, we have now demonstrated that the PBD Dimer SJG-136 and the C8-conjugated PBD Monomer GWL-78 can covalently bond to a terminal guanine of DNA, with the PBD skeleton spanning only two base pairs. Control experiments with the non-C8-conjugated anthramycin along with molecular dynamics simulations suggest that the C8-substituent of a PBD Monomer, or one-half of a PBD Dimer, may provide stability for the adduct. This observation highlights the importance of PBD C8-substituents, and also suggests that PBDs may bind to terminal guanines within stretches of DNA in cells, thus representing a potentially novel mechanism of action at the end of DNA strand breaks.

## Introduction

**Fig 1:**
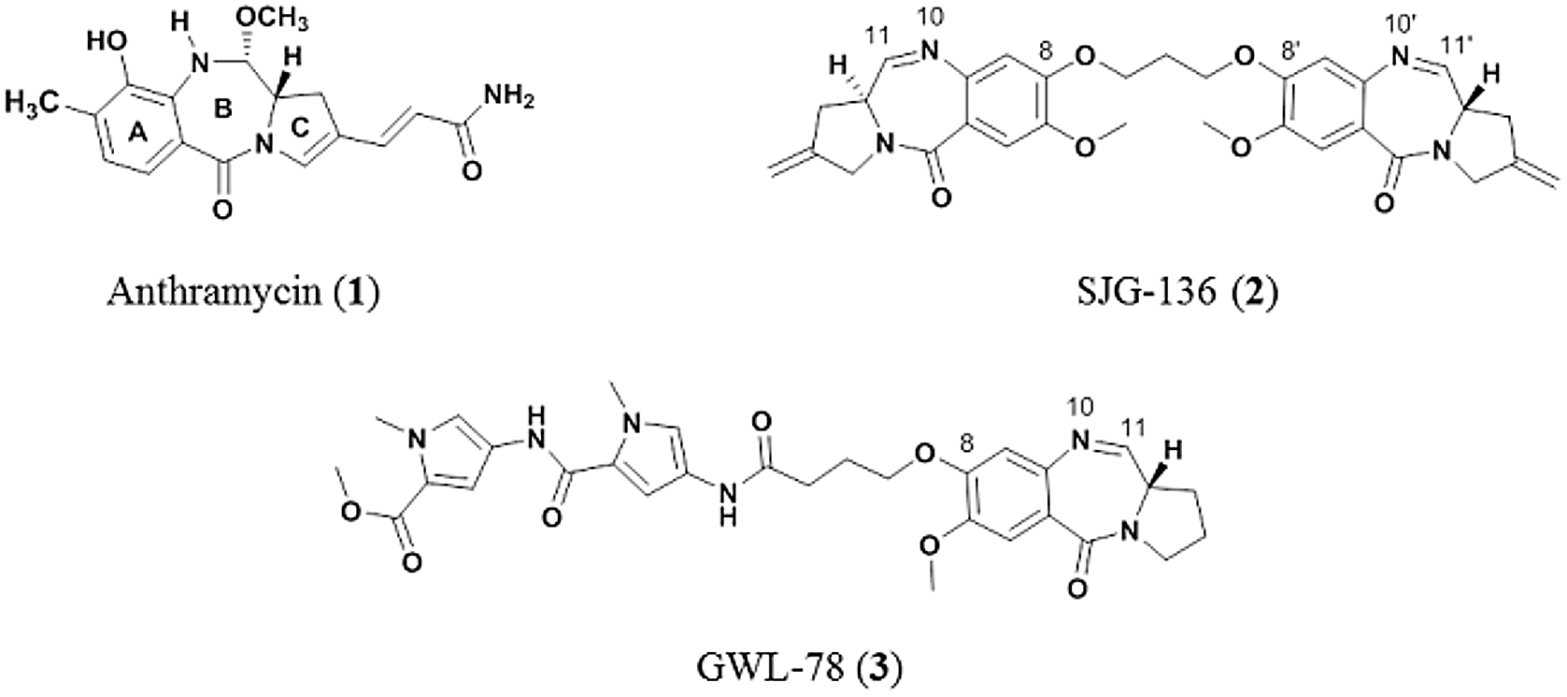
Structures of PBDs used in this study. The naturally occurring anthramycin as its C11-methyl ether (**1**), the synthetic PBD Dimer SJG-136 (**2**), and the synthetic C8-*bis*-pyrrole PBD Conjugate GWL-78 (**3**).

The pyrrolo[2,1-*c*][1,4]benzodiazepines (PBDs) are a family of sequence-selective DNA minor-groove binding agents [1–5] which are growing in importance due to their use as payloads in Antibody-Drug Conjugates (ADCs) [6–8]. The naturally occurring PBDs produced by *Streptomyces* and *Micrococcus* species are monomeric (*e*.*g*., anthramycin **1**, Fig 1) and form singly-alkylated DNA-adducts, whereas the synthetic PBD Dimers consist of two PBD units joined through a C8/C8′-linker and can form interstrand or intrastrand DNA cross-links in addition to mono-adducts [9–12]. One PBD Dimer, SJG-136 (**2**, Fig 1), successfully completed Phase I clinical trials [13–15], and reached Phase II evaluation in ovarian and hematological cancers. The DNA-binding affinity and cytotoxicity of PBD Monomers has been enhanced by attaching heterocyclic units to the C8-position (*e*.*g*., GWL-78, **3**, Fig 1), and molecules of this type have been shown to inhibit the binding of certain transcription factors to their consensus DNA sequences [16]. PBD molecules have a chiral center (*S*) at their C11a-position which provides them with the appropriate 3-dimensional shape to fit perfectly within the DNA minor-groove [4]. They also possess an electrophilic N10-C11 moiety (*i*.*e*., interconvertible imine, carbinolamine or carbinolamine methyl ether functionalities) that can form a reversible covalent aminal bond between their C11-position and the nucleophilic C2-NH_2_ group of a guanine base [4, 17–19]. PBD Monomers such as anthramycin (**1**) typically span three base pairs of DNA with a reported preference for 5′-Pu-G-Pu-3′sequences [4, 20], although more recent data suggest that they have a kinetic preference for 5′-Py-G-Py-3′ sequences [21]. Reports in the literature based on NMR [22], X-ray crystallography [23], molecular modeling [24], gel-based experiments (*e*.*g*., DNA footprinting [25] and *in vitro* polymerase stop assays [26]) suggest that PBDs have a thermodynamic rank order of preference for binding to 5′-Pu-G-Pu-3′ > Pu-G-Py ~ Py-G-Pu > Py-G-Py sequences, with the adducts oriented so that the PBD A-ring points towards the 3′-terminus of the covalently-modified strand. The covalent binding of PBDs to DNA is presumed to be a two-step process [27], the first involving recognition of a favoured low-energy binding site by fast, reversible non-covalent association of the drug in the minor groove through interactions including hydrogen-bonding, Van der Waals and electrostatic contacts. If these non-covalent interactions are weak, the molecule presumably dissociates and then re-associates at another site, with this process repeating itself until it finds a suitable low-energy triplet with the C2-NH_2_ of the central guanine aligned for nucleophilic attack at the PBD C11-position. The second step of covalent bond formation can then occur between the guanine C2-NH_2_ and PBD, at which point the molecule is locked into position. The rate of this second covalent step is much slower than the initial non-covalent association and can, according to the literature, take up to 24 hours to complete [9].

It is known from the literature that the DNA-binding affinity of PBD molecules correlates well with their *in vitro* cytotoxicity [10, 27, 28] in tumour cell lines. As a result of their sequence-selective covalent binding to DNA, it has been shown that PBDs can mediate a number of biological effects including the inhibition of endonucleases [29], RNA polymerase [27, 30] and transcription factor binding [16, 31–35]. For the PBD Dimers, cytotoxicity is likely to result from the poor recognition of DNA damage by repair proteins resulting in the slow repair of mono-adducts and, more importantly, cross-links in the DNA minor groove [36]. Additionally, there have been reports of preferential repair of PBD adducts in healthy cells compared to tumour cells, with the repair response dependent on cell type, and the extent and duration of exposure to the agent [37]. Tumour cells are often deficient in one or more relevant DNA repair pathways, thus leading to selective cytotoxicity and an *in vivo* antitumour effect [38, 39].

The initial objective of this study was to investigate the interaction of the monomeric PBDs anthramycin (**1**) and GWL-78 (**3**), and the PBD Dimer SJG-136 (**2**), with transcription factor recognition sequences, and to identify the reactive guanine(s) within these sequences. For this purpose we obtained synthetic transcription factor recognition sequences with guanines located at different positions within DNA hairpin and duplex structures. These guanines were consecutively replaced with inosine bases to remove the nucleophilic C2-NH_2_ functionalities to prevent covalent interaction. Through this process, we made the surprising observation that **2** and **3** can covalently bond to terminal guanine residues, a previously unobserved phenomenon. This has possible consequences for the biological mechanism of action of C8-substituted PBD Monomers and Dimers, as it means that they may be able to bind to the ends of strand breaks in the DNA of cells.

## Materials and Methods

### Single-Stranded and Hairpin Oligonucleotides

Single-stranded (SS) and hairpin oligonucleotides were purchased from Eurogentec (Southampton, UK) in lyophilized form. They were annealed to form double-stranded (DS) DNA according to the procedures described below.

### Annealed Double-Stranded Oligonucleotides

Each single-stranded oligonucleotide was dissolved in 1:1 of annealing buffer (10 mM Tris-HCl pH 8.5/50 mM sodium chloride/1mM EDTA) and 100 mM ammonium acetate to form stock solutions of 1 mM. Solutions of DS DNA were prepared by heating to 85°C for 10 mins in a heating/cooling block (Grant Bio, UK), and then allowed to cool to room temperature followed by storage at -20°C overnight to ensure completion of the annealing process. Working solutions of double-stranded oligonucleotides of 25 μM were prepared by diluting stored stock solutions with 20 mM ammonium acetate (Sigma-Aldrich, UK) followed by storage at -20°C.

### Annealed Hairpin Oligonucleotides

Each hairpin-forming single-stranded oligonucleotide was dissolved in annealing buffer (10 mM Tris-HCl pH 8.5/50 mM sodium chloride/1 mM EDTA in a 1:1 ratio) to form stock solutions of 1 mM. For hairpin formation, solutions were heated to 85°C for 10 min in a heating/cooling block (Grant Bio, UK), and then allowed to cool slowly to room temperature followed by storage at -20°C overnight for completion of the annealing process. Working solutions of hairpin oligonucleotides of 25 μM were prepared by diluting stored stock solutions with 100 mM ammonium acetate (Sigma-Aldrich, UK) followed by storage at -20°C. Hairpin oligonucleotides were designed (*i*.*e*., 19 bases in length) to ensure the presence of a DNA minor-groove environment necessary for covalent binding of a PBD, rather than for any particular biological relevance.

### Preparing Solutions of Anthramycin (1), SJG-136 (2) and GWL-78 (3)

PBDs **1**, **2** and **3** were dissolved in dimethyl sulfoxide (DMSO anhydrous, ≥ 99.9%) to form stock solutions of 10 mM which were stored at -20°C. Working solutions of each PBD of 100 μM were prepared by diluting stock solutions with nuclease-free water (Biology Grade, Fisher Scientific, UK), and these were stored at -20°C for not more than two weeks, and thawed to room temperature when required.

### Preparation of PBD/DNA Complexes

PBD/DNA complexes were prepared by adding one of the PBD working solutions (100 μM) to an annealed hairpin or double-stranded oligonucleotide working solution (25 μM) in a 4:1 ratio (PBD/DNA). The mixture was agitated for 5–10 seconds using a vortex mixer, and then incubated for 24 hours at 25°C before subjecting to ion-pair reversed-phase HPLC and mass spectrometry analysis.

### Ion-Pair Reversed-Phase HPLC Analysis

Liquid chromatography was performed on a Thermo Scientific UltiMate 3000 system equipped with a 2.1 x 50 mm XBridge^™^ OST C18 column packed with 2.5 μm particles (Waters Ltd., UK), using Chromeleon 7 software (Version 7.1.1.1127). The gradient system used for LC analysis consisted of 100 mM triethylammonium bicarbonate (TEAB) as Buffer A and 40% acetonitrile (ACN) in water (HPLC grade, Fisher Scientific, UK) as Buffer B. For Buffer A, a 1 M pre-formulated TEAB solution was purchased from Sigma-Aldrich (UK), and diluted to the required concentration with HPLC grade water. The gradient was ramped from 90% A at 0 min to 55% A at 18 min, then to 20% A at 22 min, and finally to 10% A at 23.5 min. UV absorbance was monitored at 254 nm.

### Mass Spectrometric Analysis (MALDI-TOF-MS)

Reaction mixtures of anthramycin (**1**), SJG-136 (**2**) and GWL-78 (**3**) with hairpin or double-stranded oligonucleotides were prepared and incubated as described above. Drug/DNA adduct samples were diluted with 0.1 M triethylammonium acetate buffer (TEAA, Sigma-Aldrich, UK) in 1:1, 1:4, and 1:10 ratios. The matrix was comprised of 50 mg 3-hydroxypicolinic acid (HPA) in 1 mL 50% acetonitrile (ACN) and 50 mg ammonium citrate dibasic (DAC) in 1 mL water mixed in a 9:1 ratio (HPA/DAC). 1 μL of drug/DNA was mixed with 1 μL matrix, and 0.2 μL was then spotted onto the MALDI target plate and allowed to dry. Analysis was carried out using a Bruker Daltonics Autoflex^™^ automated high-throughput MALDI-TOF-MS system with nitrogen laser in positive linear mode using delayed extraction (500 ns) and an accelerating voltage of 25000 V. Acquisition was between 1000–10000 Daltons with 100 shots per spectrum.

### Mass Spectrometric Analysis (LC-ESI-MS/MS)

Chromatographic separation of DNA and SJG-136/DNA adduct complexes was performed on a Agilent 1200 HPLC system utilising a XBridge 50 x 2.1 mm C18 column packed with 2.5 μm particles (Waters, UK). Mobile phase A consisted of 50 mM TEAB in water pH 7.4, and mobile phase B consisted of 40% acetonitrile in water. After 1 min at 10% B, the proportion of B was raised to 80% over the next 18 min, followed by an increase to 100% B over the next 4 min, maintained at 100% B for a further 1.5 min, before returning to 10% B in 6 seconds and re-equilibration for a further 3 min 54 seconds, providing a total run time of 28 min. The flow rate was maintained at 300 μL/min and the eluent directed to the Dual-ESI source of an Agilent 6510 Q-TOF mass spectrometer. Ions from the Dual-ESI source, operated in the negative ESI mode, were transmitted to the pusher of the Q-TOF in either MS or MS/MS mode. The full mass range was set from *m/z* 300 to 5,000 for MS mode with an acquisition rate of 0.58 and time of 1716.8 corresponding to transient/spectrum of 9652.

For LC-MS/MS analysis of the Hairpin oligonucleotides, each sample (25 μM in 100 mM ammonium acetate) was injected (20 μL) onto the reversed-phase column and eluted into the Q-TOF at a flow-rate of 300 μL/min. Dual-ESI parameters were as follows: gas temperature, 350°C; drying gas, 10 L/min; nebuliser, 50 psig; VCap, 3500; fragmentor, 20 V; skimmer, 65 and OCT1 RFVpp, 750. The acquisition rate was 9652 transients/spectrum for MS and MS/MS mode. Mass spectra were acquired in profile and centroid mode. The Q-TOF was calibrated continuously using a lock-mass calibration mixture during analytical runs, and mass accuracy was in all cases better than 2 ppm. The Q-TOF was operated at a resolution of 30,000.

For the analysis of SJG-136/DNA adduct samples, 20 μL of the reaction mixture was injected onto the C18 column. MS/MS spectra were recorded in a targeted MS/MS acquisition mode with the mass range set from *m/z* 100 to 4000, with an acquisition rate of 0.58 and a time of 1716.8 corresponding to a transient/spectrum of 9652. The targeted list was set for [M-3H]^3-^ ions corresponding to DNA or SJG-136/DNA determined from the full MS mass spectra. For the acquisition of MS/MS spectra, the collision energy was set to 20, 30 and 40 to determine the maximum number of MS/MS fragments. The MS/MS isolation width was set to medium 4 *m/z* to allow the selection of mono-isotopic precursor ions.

### Molecular Modelling and Molecular Dynamics Simulations

Hairpin and duplex DNA sequences used in this study (including the TTT-loop regions) were constructed using the AMBER module *nab* [40]. When present, the TTT-loop was covalently linked to the backbone of the DNA using *xleap*, and parameters derived in-house. Inosine-containing sequences were created through the deletion of the exocyclic amine groups of guanine residues. Anthramycin (**1**), SJG-136 (**2**) and GWL-78 (**3**) were docked in the minor groove using the AMBER module *xleap*, in which *parm99SB*, modified *parmbsc0* [41] and Gaff AMBER force field parameters were loaded. *Antechamber* was used to construct.*mol2* files through the addition of Gasteiger charges, and missing parameters were generated using *parmchk*. A covalent bond was generated between the exocyclic amine groups of selected guanines (guided by molecular mechanics calculations [42]), to form either mono-alkylated or inter/intra-strand cross-linked adducts. Energy minimisation was then undertaken in a gradient manner by initially placing the DNA under a high force constraint (*i*.*e*., 500 kcal mol^-1^ Angstrom^-2^), which was then reduced in stages to zero to enable the PBD molecule to find its local energy minimum, followed by reduction in force in a periodic manner with a relaxation of restraints. Production simulations in implicit solvent (GBSA) were run for a period of 10 ns, and atomic coordinates were saved at 1 ps intervals. A *ptraj* script was used to ascertain the lowest potential energy derived during adduct simulations, and extended simulations (50 ns in duration) were also undertaken on selected ligand:DNA adducts to show that increasing simulation time did not have any effect on differences in potential energies (data presented in S27 Fig). Hydrogen-bonding analysis of molecular dynamics simulations was undertaken using VMD [43]. All models were created using Chimera [44].

## Results

**Fig 2:**
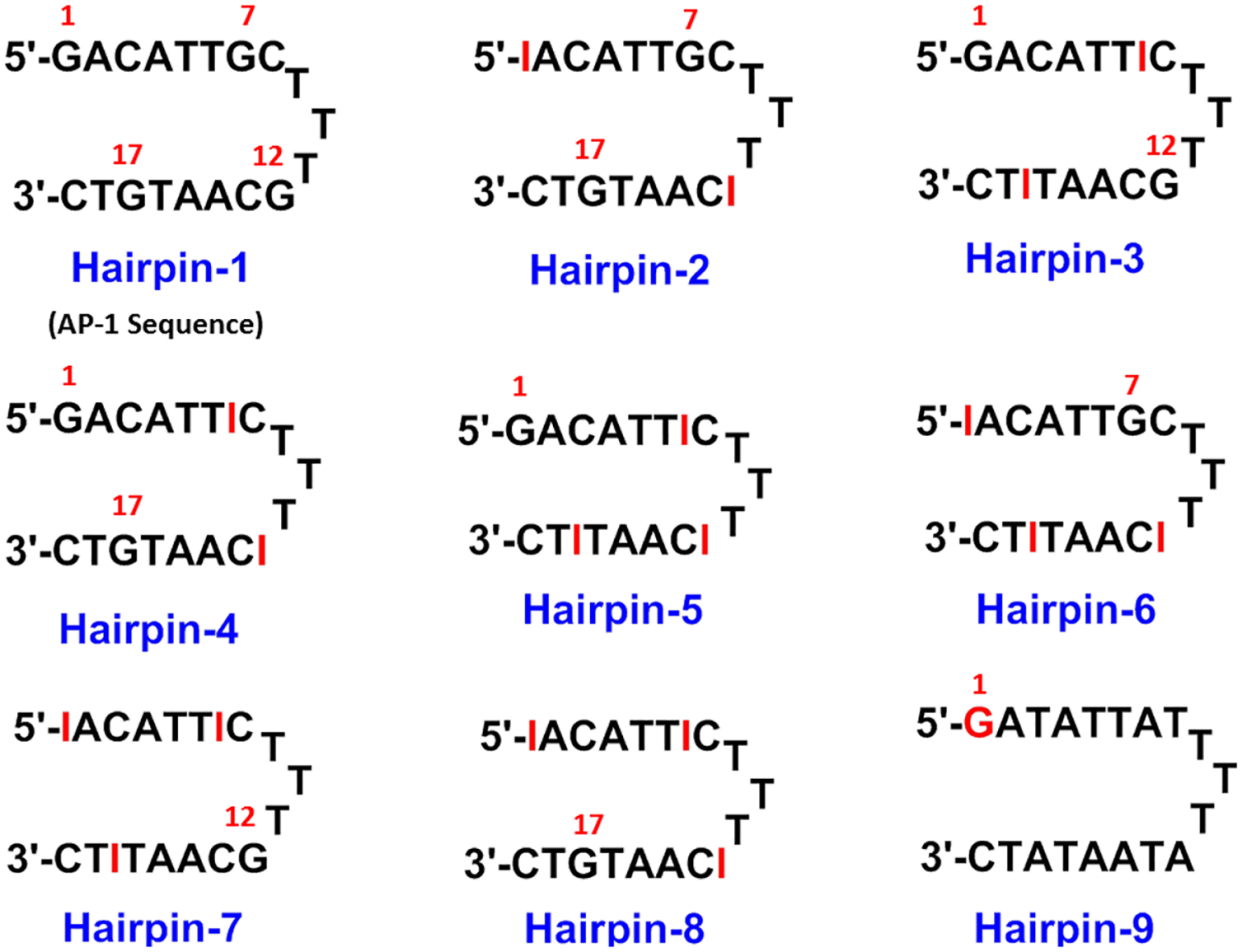
Structures of Hairpins 1–9 used in this study. Hairpin-1, the AP-1 transcription factor recognition sequence; Hairpins 2–4, the same AP-1 sequence but with two of the four guanines replaced with inosines in each case to study cross-linking; Hairpins 5–8, the same AP-1 sequence but with three of the four guanines replaced with inosines in each case to study mono-alkylation; Hairpin 9, the same AP-1 sequence but with three of the four guanine bases (except the 5’-terminal-guanine) mutated to an A.

**Fig 3:**
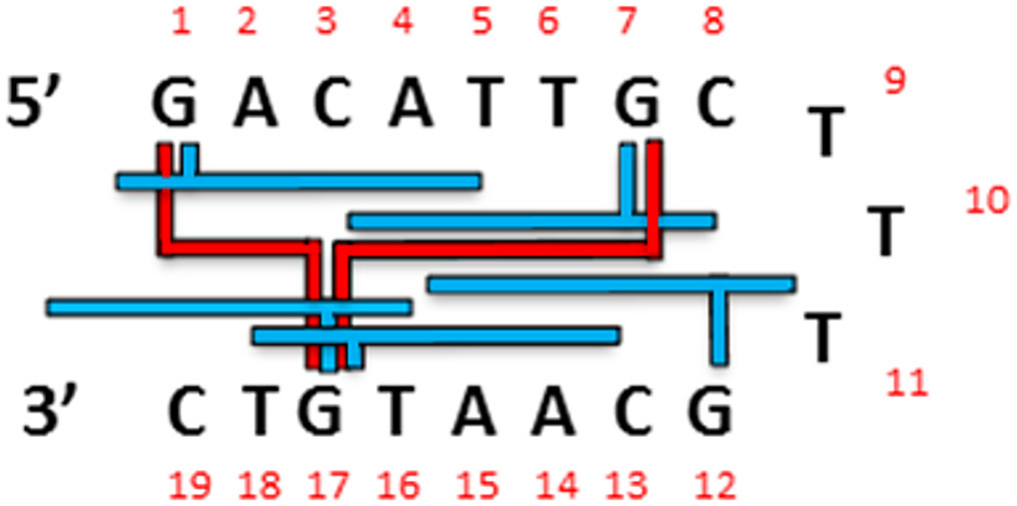
AP-1 consensus sequence. Schematic diagram of the various possible mono-alkylated (blue) and interstrand cross-linked (red) adducts that could potentially form between SJG-136 (**2**) and the parent AP-1 sequence (Hairpin-1).

During a study of the interaction of SJG-136 (**2**) with various DNA transcription factor recognition sequences [33], we observed that it rapidly formed an adduct with the AP-1 hairpin DNA sequence (Hairpin-1, Fig 2), with a major new peak appearing in the chromatogram at retention time (RT) 7.53 min after 24 hours of incubation, and with reaction complete by 24 hours (S1A and S1B Fig). The stoichiometry of the adduct was confirmed as 1:1 SJG-136/Hairpin-1 by MALDI-TOF-MS with an observed mass of 6351.2 m/z (theoretical mass: 6350.41 m/z) (S1C Fig). Given that we had previously shown [45] that **2** can form mono-adducts, and intra- and interstrand cross-links of different lengths according to the following rank order of preference: Pu-GAATG-Py > Pu-GATC-Py >> Pu- GATG-Py and Pu-GAATC-Py [9], we initially assumed that the adduct formed was most likely to be either the extended G7-G17 3’-GTAAC-5’ interstrand cross-link, a G7 or G17 mono-alkylated adduct, or a combination of one or more of these (Fig 3). In order to investigate this further, we designed the DNA Hairpins 2–4 (Fig 2) which are based on the same AP-1 sequence but with each hairpin having two of its four guanines replaced with non-nucleophilic inosines (*i*.*e*., guanine residues without nucleophilic C2-NH_2_ groups), still allowing the possibility of interstrand cross-link formation. We also designed Hairpins 5–8 which have three of their four guanines replaced with inosines, thus leaving only one reactive guanine (*i*.*e*., G1, G7, G12 or G17) in each case, to study monoalkylation.

### Interaction of SJG-136 (2) with the Inosine-Modified AP-1 Hairpin Oligonucleotides (Hairpins 2–8)

The substitution patterns of Hairpins 2–4 (Fig 2) allowed the possibility of interstrand cross-linking at G7 and G17 (Hairpin-2), G1 and G12 (Hairpin-3) and G1 and G17 (Hairpin-4). HPLC analysis of annealed Hairpins-2-4, showed single peaks for the hairpin sequences with retention times ranging from 6.78 minutes to 7.44 min (Table 1). The hairpin sequences provided correct m/z values within a narrow range of 5764.1 to 5764.5 by MALDI-TOF-MS (Table 1). Hairpins-2-4 were incubated with **2** at 25°C for 24 hours in a 4:1 ratio, and new adduct peaks were observed at retention times 8.40 min, 8.08 min and 8.04 min, respectively (Table 1). In the case of Hairpin-2, a complete disappearance of the hairpin DNA peak was observed (S2A and S2B Fig) while 87% and 89% conversion to adducts was observed with Hairpin-3 (S3A and S3B Fig) and Hairpin-4 (S4A and S4B Fig), respectively. The stoichiometry of the adducts was confirmed by MALDI-TOF-MS as 1:1 **2**/Hairpin DNA on the basis of observed masses (Table 1). The formation of an adduct with Hairpin-3 was a surprising result, as a PBD had not previously been observed to bond to a terminal guanine (*i*.*e*., G1), and G12 was considered to be too close to the TTT-loop to covalently bond to a PBD. Furthermore, an interstrand cross-link between G1 and G12 would be too long to form. Therefore, our conclusion was that, for Hairpin-3, **2** was bonding to the terminal G1 *via* one PBD unit, with the second PBD unit pointing towards the TTT-loop (Fig 3).

**Table 1:** Summary of HPLC/MALDI-TOF results obtained on the interaction of SJG-136 with Hairpins 1–8.

DNA Sequence	RT DNA (min)	RT 1:1 SJG-136/DNA Adduct (min)	Adduct Formation After 24 Hours (%)	Observed Mass DNA (kDa)	Theoretical Mass DNA (kDa)	Observed Mass 1:1 SJG-136/DNA Adduct (kDa)	Theoretical Mass 1:1 SJG-136/DNA Adduct (kDa)
**Hairpin-1**	7.31	7.53	100	5794.8	5793.8	6351.2	6350.4
**Hairpin-2**	6.78	8.40	100	5764.5	5763.8	6319.5	6320.4
**Hairpin-3**	7.43	8.08	87	5764.2	5763.8	6321.0	6320.4
**Hairpin-4**	7.44	8.04	89	5764.1	5763.8	6320.9	6320.4
**Hairpin-5**	7.64	14.03	50	5748.3	5748.8	6305.1	6305.4
**Hairpin-6**	8.01	9.24	50	5749.2	5748.8	6306.1	6305.4
**Hairpin-7**	8.14	9.07	80	5748.6	5748.8	6305.0	6305.4
**Hairpin-8**	8.03	8.86	70	5748.9	5748.8	6305.6	6305.4

**Fig 4:**
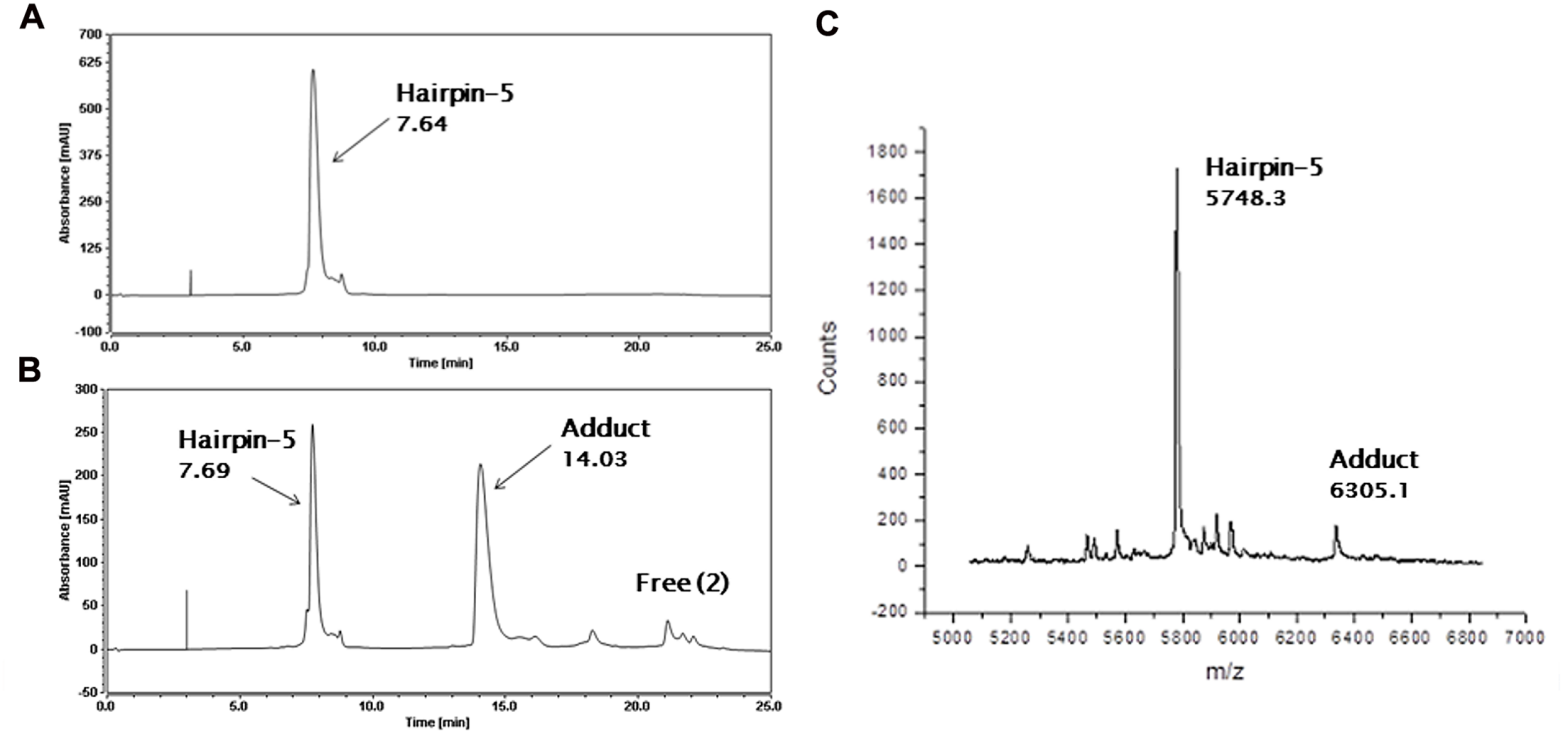
HPLC/MS data obtained from interaction of SJG-136 (2) with Hairpin-5. **A**, HPLC chromatogram showing the annealed Hairpin-5 (Fig 2) alone at RT 7.64 min; **B**, HPLC chromatogram after incubation of annealed Hairpin-5 with **2** for 24 hours, showing approximately 50% conversion to the adduct peak at RT 14.03 min; **C**, MALDI-TOF spectrum of the adduct at RT 14.03 min from Chromatogram B above. Observed mass of 1:1 **2**/Hairpin-5 adduct: 6305.1 m/z (theoretical mass: 6305.41 m/z), observed mass of DNA Hairpin-5 alone from Chromatogram **A**: 5748.3 m/z (theoretical mass: 5748.8 m/z).

In order to study mono-adduct distribution using the same HPLC/MS methodology, a further four AP-1 hairpins (Hairpin 5–8) were designed, based on the same AP-1 sequence but with a different three of the four guanines replaced with inosines in each case to leave just one reactive guanine (*i*.*e*., G1, G7, G12 or G17 in Hairpins 5, 6, 7 and 8, respectively) (Fig 2). HPLC analysis of annealed Hairpin-5, with only the terminal guanine (*i*.*e*., G1) available gave a single peak at RT 7.64 min (Fig 4A) which provided the correct m/z (5748.30) for this oligonucleotide by MALDI-TOF-MS. Following a 24 hour incubation with **2** at 25°C in a 4:1 molar ratio (**2**/DNA), a new peak appeared in the HPLC chromatogram at RT 14.03 min (Fig 4B). The adduct stoichiometry was confirmed by MALDI-TOF-MS as 1:1 **2**/Hairpin-5 based on an observed mass of 6305.1 m/z (theoretical mass: 6305.41 m/z) (Fig 4C). Although the reaction had not gone to completion by 24 hours (*i*.*e*., approximately 50% reacted), this result confirmed that a PBD unit could bond to a terminal G1 base, with the body of the covalently-attached PBD spanning two rather than three base pairs.

Similar HPLC analysis of Hairpins 6 to 8 (Fig 2) showed single peaks for the hairpin sequences with retention times ranging from 8.01 to 8.14 minutes (Table 1, S5A, S6A and S7A Figs). The hairpin sequences provided correct m/z values by MALDI-TOF-MS with masses ranging from 5748.3 to 5749.2 in close agreement with their theoretical values (Table 1). Incubation of **2** at 25°C for 24 hours in a 4:1 ratio with these hairpin sequences gave new adduct peaks at retention times 9.24, 9.07 and 8.86 minutes, respectively (Table 1, S5B, S6B and S7B Figs). In all cases the 1:1 stoichiometry of the adduct (**2**/DNA) was confirmed by MALDI-TOF-MS (Table 1, S5C, S6C and S7C Figs). Interestingly, significant differences in the extent of adduct formation were observed with these inosine-modified sequences (Table 1). In the case of Hairpin-6, the adduct was thought to be a mono-alkylated adduct with **2** bonded to G7, and with the bulk of the PBD Dimer lying in the minor groove pointing away from the loop. The approximately 50% conversion within 24 hours was assumed to reflect the lower stability of a mono- rather than a cross-linked adduct. In the case of Hairpin-7, the reaction was approximately 80% complete, also consistent with a lower rate of reaction for mono-adduct formation. This result was also surprising due to the proximity of G12 to the loop structure. Molecular Dynamics Simulations (see later) suggested that, although located close to the TTT-loop, a **2**:G12 mono-alkylated adduct is feasible, with the second PBD unit of the dimer orientated away from the loop and forming non-covalent interactions in the minor groove. Finally, reaction with Hairpin-8 was 70% complete after 24 hours with one of the PBD units presumably bonding to G17 with the bulk of the molecule lying in the minor groove pointing toward the loop.

The reactivity of G1 was particularly surprising, as it means that the covalently bonded PBD unit must span only two base pairs, a phenomenon not previously observed or thought to be possible. Previous studies on the DNA binding characteristics of PBDs have suggested that a minimum of three consecutive DNA bases with a central covalently-reacting guanine (*i*.*e*., Pu-G-Pu) are required for covalent attachment [20]. To investigate this further we carried out an LC-MS/MS analysis of the **2**/Hairpin-5 adduct. A base peak ESI-MS chromatogram of Hairpin-5 is shown in S8 Fig, and a full scan ESI mass spectrum in S9 Fig for the chromatographic peak eluting at 7.1 min in this experiment (S8 Fig). A major signal at *m/z* 1914.9 corresponding to the triply charged [M-3H]^3-^ ion, and a minor signal at *m/z* 2873.1 corresponding to the doubly charged [M-2H]^2-^ ion were observed. A full ESI mass spectrum of the expanded mass range from *m/z* 1910.0 to 1919.0 provided additional information on the charge state of this triply charged species (S10 Fig), showing a mass difference of 0.34 between *m/z* 1914.3217 and 1914.6622, thus confirming the triply charged [M-3H]^3-^ ion of Hairpin-5 under the ESI conditions. MS/MS spectra were acquired at collision energies of 20, 35 and 40, and S11, S12, S13 and S14 Figs show MS/MS spectra of *m/z* 1914 at these various collision energies. The MS/MS spectrum at collision energy 35 generated a reasonable number of fragment ions (S12 Fig). The *m/z* theoretical values of the collision-induced dissociation (CID) were calculated by Mongo Oligo Mass Calculator v2.06, and were compared to the experimental *m/z* values of fragment ions. A predicted CID fragmentation pattern of Hairpin-5 is shown in the insert to S12 Fig. Cleavage of the phosphodiester bond leads to fragment-ions containing the 5’-OH group which are symbolised by a_n_, and those containing the 3’-OH group by W_n_. The major fragment ion at collision energy 40 is at *m/z* 610 and corresponds to W_18._ The cleavage of the nucleic acid chain yields fragment-ions of (a_n_-B_n_) characteristic of the sequence from 5’- to 3’-, and the MS/MS fragment-ions at *m/z* 407.4 and 739.1 correspond to (a_10_-T) and (a_3_-C), respectively, thereby indicating a 5’-OH group phosphodiester fragmentation (S14 Fig).

**Fig 5:**
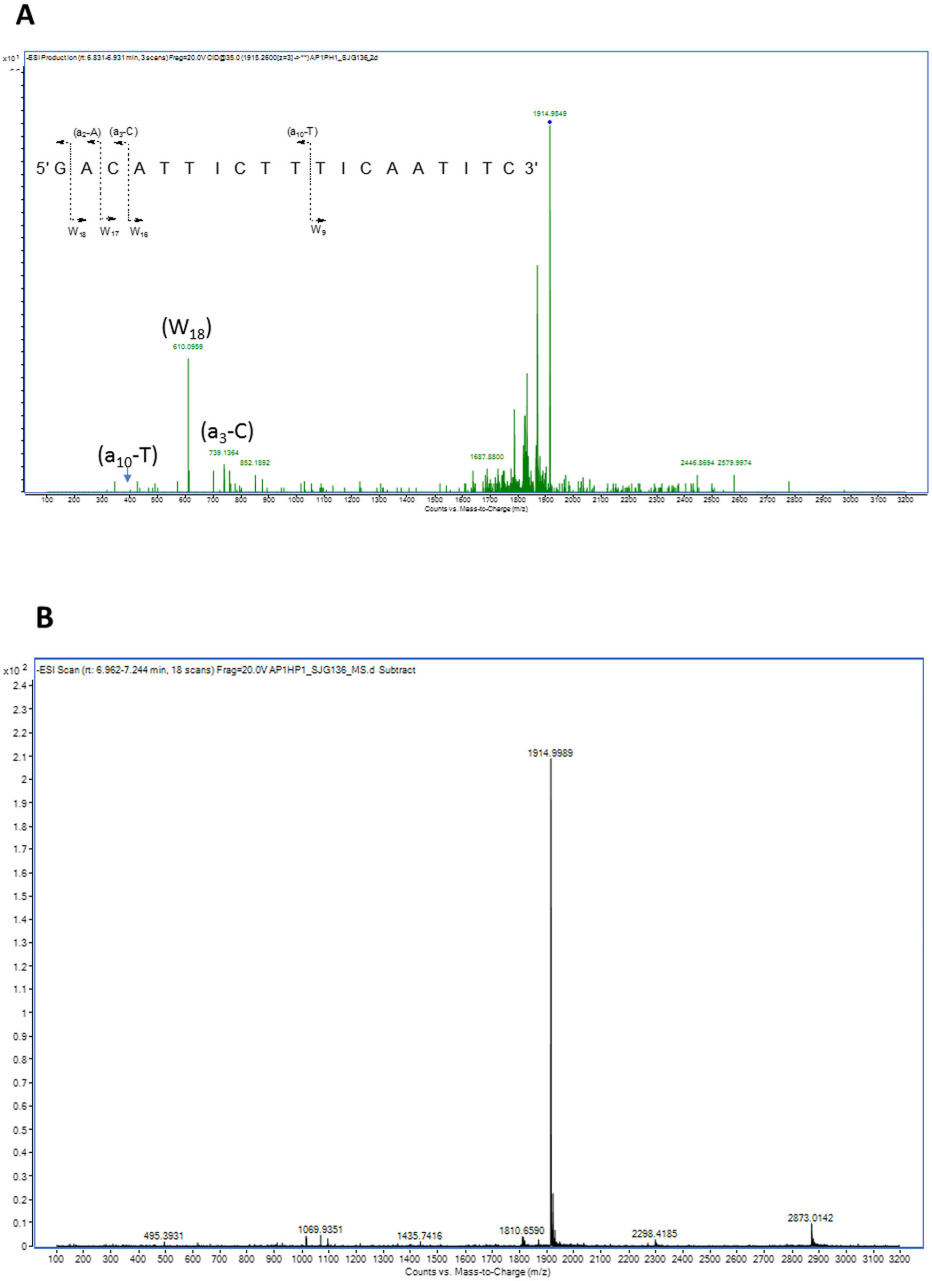
**A**, Full ESI mass spectrum for the chromatographic peak eluting at 9.1 min corresponding to the **2**/Hairpin-5 adduct. The signal at *m/z* 2100.4 corresponds to the triply charged [M-3H]^3-^ ion of the adduct; **B**, The MS/MS spectrum of the *m/z* 2100.4 ion from the full ESI spectrum in **A** at a collision energy of 35.

S15 Fig shows an ion pair reversed-phase reconstructed ion chromatogram (RIC) for *m/z* 1914 of the SJG-136/DNA mixture at a 4:1 molar ratio after 12 h incubation. Two major chromatographic peaks were detected. The chromatographic peak at 7.1 min corresponds to Hairpin-5 itself, and the new peak at 9.1 min corresponds to the SJG-136/Hairpin-5 adduct which was confirmed by the full ESI mass spectrum shown in Fig 5A and S16 Fig. The peak at *m/z* 2100.4 corresponds to the [M-3H]^3-^ ion of SJG-136/Hairpin-5 (5751.96+556.61 Da), thereby representing the molecular weight of the adduct (6308.57 Da). We also observed an in-source fragmentation under ESI conditions that generate an additional peak at *m/z* 1915.3 corresponding to Hairpin-5. The MS/MS spectrum of the *m/z* 2100.4 ion at a collision energy of 35 is shown in Fig 5B. The location of SJG-136 attached to the 5’-OH-guanine was elucidated by the presence of the peak at *m/z* 964 characteristic of the (a_10_-T) fragment-ion. The peak at *m/z* 407.4 corresponded to the (a_10_-T) fragment-ion of Hairpin-5, whereas the peak at *m/z* 964 corresponded to the (a_10_-T) fragment-ion of Hairpin-5+SJG136 (407.4+556.6 Da).

In summary, the results from the experiments with Hairpins 5–8 demonstrated that all four guanines (*i*.*e*., G1, G7, G12 or G17) are reactive toward **2**, and are capable of forming mono-alkylated adducts.

### Interaction of Anthramycin (1) and GWL-78 (3) with the Inosine-Modified AP-1 Hairpin Sequences

To determine whether the size of the PBD molecule might affect the mode of binding to the AP-1 hairpins, similar experiments were carried out with anthramycin (**1**) and the PBD C8-conjugate GWL-78 (**3**). First, **1** was incubated with Hairpin-5 (with only the 5’-terminal guanine available for covalent binding) in a 4:1 ratio (**1**/DNA) for 24 hours at 25°C. However, no changes were observed in the HPLC chromatogram, with the unreacted Hairpin-5 peak at RT 7.90 min remaining the major peak (S17A and S17B Fig) as confirmed by MALDI-TOF-MS (observed mass: 5748.0 m/z, theoretical mass: 5748.8 m/z) (S17C Fig). This suggested that substitution at the C8-position, as in SJG-136 (**2**) may have a significant influence on the binding properties of a PBD by enhancing interaction of the molecule in the DNA minor groove through non-covalent interactions (*e*.*g*., van der Waals, hydrogen bonds and/or electrostatic interactions).

The next step was to study GWL-78 (**3**), a synthetic PBD Monomer substituted at the C8-position with a *bis*-pyrrole side-chain that allows the molecule to span a total of five DNA base pairs. It was anticipated that this molecule may behave more like SJG-136 (**2**) than anthramycin (**1**) due to the large non-covalent DNA-binding moiety at the C8-position. As predicted, when **3** was incubated with Hairpin-5 (RT 8.69 min, S17D Fig) in a 4:1 ratio (**3**/DNA) for 24 hours at 25°C, a new peak was observed at 11.13 min (S17E Fig), although the extent of reaction was much lower compared to **2**. The new peak was identified as the 1:1 **3**/Hairpin-5 adduct by MALDI-TOF-MS (observed mass: 6338.6 m/z, theoretical mass: 6339.4 m/z) (S17F Fig).

From these results it was concluded that covalent bonding of a PBD to a terminal guanine may only occur when there is sufficient molecular bulk at the C8-position (*e*.*g*., another PBD unit in the case of SJG-136, or a *bis*-pyrrole unit in the case of GWL-78) to provide stabilization to the adduct by interacting non-covalently in the adjacent minor groove.

### Interaction of Anthramycin (1), SJG-136 (2) and GWL-78 (3) with Hairpin-9

**Fig 6:**
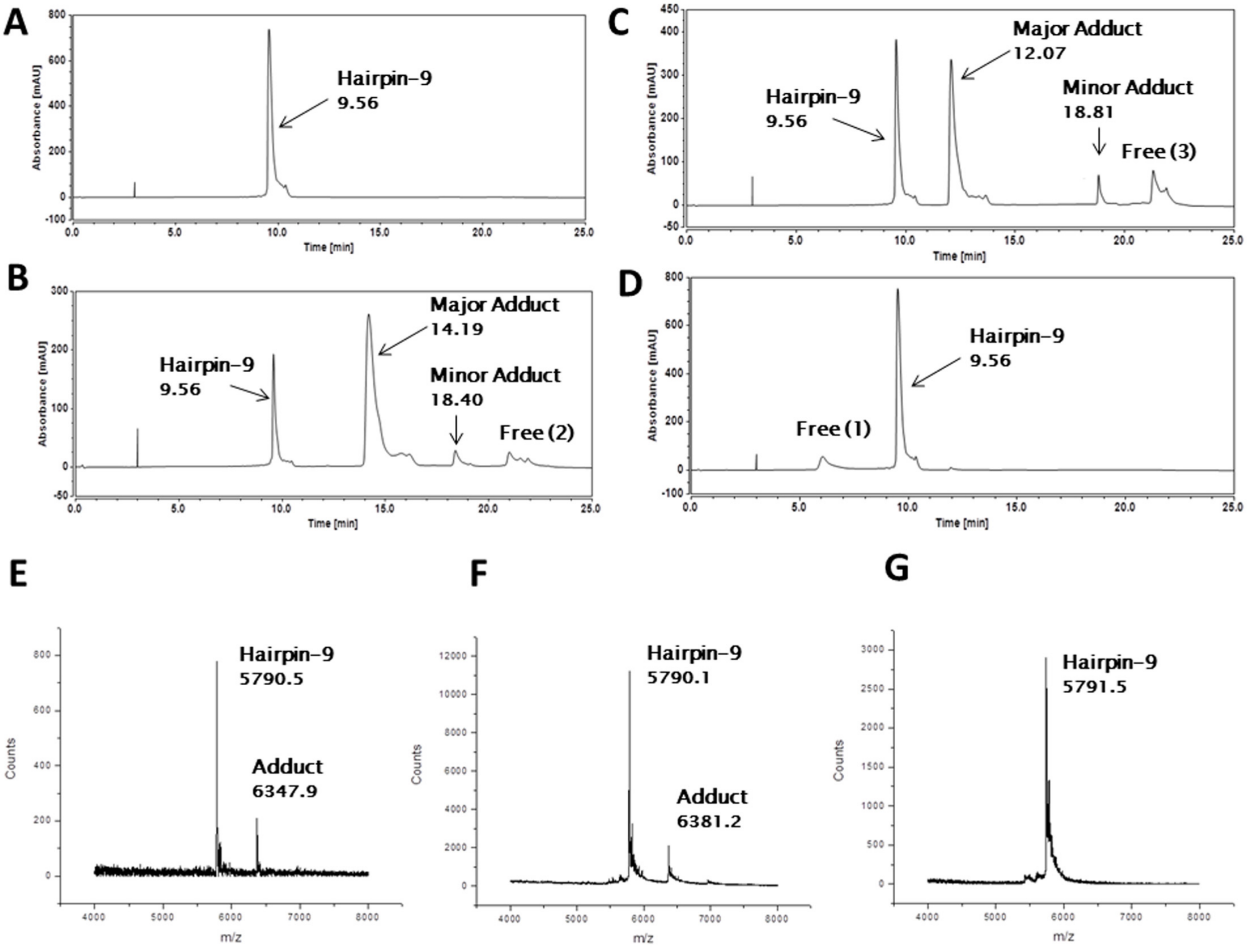
HPLC/MS data obtained from interaction of SJG-136 (2), GWL-78 (3) and anthramycin (1) with Hairpin-9. **A**, Annealed Hairpin-9 at RT 9.56 min; **B**, Annealed Hairpin-9 after incubating with **2** for 24 hours with the appearance of one new major adduct peak at RT 14.19 and a minor peak at RT 18.40 min; **C**, Annealed Hairpin-9 after incubating with 3 for 24 hours with the appearance of one new major adduct peak at RT 12.07 and a minor peak at RT 18.81 min; **D**, Annealed Hairpin-9 after incubating with **1** for 24 hours showing no adduct formation after 24 hours; **E**, MALDI-TOF spectrum of **2** with Hairpin-9 confirming the identity of adduct formation, Hairpin-9 observed mass: 5790.5 m/z (theoretical mass: 5790.9 m/z), **2**/Hairpin-9 adduct observed mass: 6347.9 m/z (theoretical mass: 6347.5 m/z); **F**, GWL-78 (**3**) with Hairpin-9 confirming the stoichiometry of adduct formation, Hairpin-9 observed mass: 5790.1 m/z (theoretical mass: 5790.9 m/z), **3**/Hairpin-9 adduct observed mass: 6381.2 m/z (theoretical mass: 6381.5 m/z); **G**, Anthramycin (**1**) with Hairpin-9 confirming no adduct formation, Hairpin-9 observed mass: 5791.5 m/z (theoretical mass: 5790.9 m/z).

In order to rule out whether these new observations of a PBD Monomer (**3**) and Dimer (**2**) bonding to a terminal guanine might be due to a change in DNA conformation caused by the insertion of inosine bases, the interaction of **1**, **2**, and **3** with Hairpin-9 (Fig 2) was studied. This hairpin contained a 5’-terminal guanine without any inosine modifications. Instead, G7, G12 and G17 were replaced with adenine bases, and their corresponding cytosines with thymines. Hairpin-9 was incubated with **1**, **2**, and **3** in a 4:1 ratio (PBD/DNA) for 24 hours at 25°C, and subjected to HPLC and MALDI-TOF-MS analysis. Hairpin-9 alone gave a single peak at RT 9.56 min (Fig 6A) identified by MALDI-TOF-MS. After reaction with **2**, one new major peak at RT 14.19 min appeared along with a minor peak at RT 18.4 min (Fig 6B). The stoichiometry of the main new peak as 1:1 **2**/Hairpin-9 was confirmed by MALDI-TOF-MS analysis with an observed mass of 6347.9 m/z (theoretical mass: 6347.5 m/z) (Fig 6E). Incubation of **3** with Hairpin-9 gave one new major peak at RT 12.07 min and a minor peak at RT 18.81 min (Fig 6C). Reaction was not complete after 24 hours with some Hairpin-9 remaining (Fig 6C). The stoichiometry of the main adduct formed was confirmed by MALDI-TOF-MS analysis as 1:1 **3**/Hairpin-9 with a mass of 6381.2 m/z (theoretical mass: 6381.5 m/z) (Fig 6F). The identity of the minor peaks at RT 18.40 min (for **2**) and RT 18.81 min (for **3**) could not be confirmed. Lastly, HPLC analysis of the interaction of **1** with Hairpin-9 provided no changes in the chromatogram (Fig 6D), with MALDI-TOF-MS (Fig 6G) confirming that no adduct had formed.

In summary, these results supported the observations made with Hairpin-5, and confirmed that the inosine mutations had not altered the reactivity of the DNA toward the PBDs.

### Interaction of SJG-136 (2) with Inosine-Modified Duplex AP-1 Oligonucleotides

In order to explore whether the observations made within the inosine-modified AP-1 hairpins (Fig 2) were a consequence of their hairpin structure, a similar series of experiments was carried out on the sequence-related DNA duplexes (S18 Fig). As with the AP-1 hairpin oligonucleotides 5–8 (Fig 2), three of the four guanines available for covalent attachment of a PBD were replaced with non-nucleophilic inosines resulting in only one guanine available for covalent bonding in each case. Each duplex oligonucleotide had a length of 8 base pairs, as it has been previously demonstrated by this laboratory that a minimum of 7 base pairs is required to ensure a DNA minor-groove environment suitable for PBD binding (*unpublished data*).

**Fig 7:**
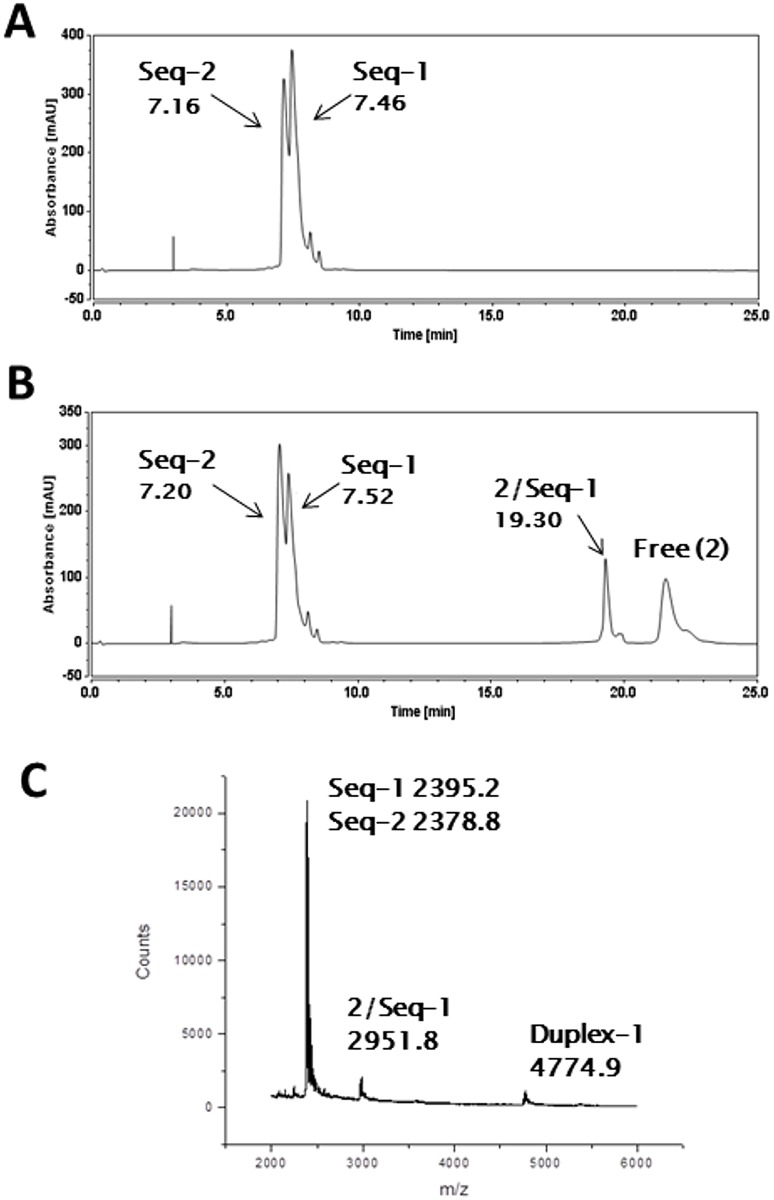
HPLC/MS data for the interaction of SJG-136 (2) with Duplex-1. **A**, Annealed Duplex-1 (S18 Fig) at RT 7.16 min (Seq-2) and RT 7.46 min (Seq-1), showing that the duplex had denatured under the HPLC conditions. **B**, Annealed Duplex-1 after incubating with **2** for 24 hours showing the appearance of one new adduct peak at RT 19.30 min (**2**/Seq-1), with the reaction incomplete after 24 hours (~13% adduct conversion); **C**, MALDI-TOF spectrum of **2** with Duplex-1, confirming the stoichiometry of the adduct formed; single strands of Duplex-1, observed masses: *Seq-2*, 2378.8 m/z and *Seq-1*, 2395.2 m/z (theoretical mass: *Seq-2* 2379.6 m/z and *Seq-1* 2394.6 m/z), and Duplex-1 observed mass: 4774.9 m/z (theoretical mass: 4774.2 m/z), **2**/*Seq-1* adduct observed mass: 2951.8 m/z (theoretical mass: 2951.21 m/z). The insignificant differences in the RT for the single-stranded oligonucleotides in chromatograms A and B are due to HPLC run-time variations.

Annealed Duplex-1 (*Seq-1*/*Seq-2*) (S18 Fig) gave two peaks in the HPLC chromatogram at RT 7.16 min (*Seq-2*) and RT 7.46 min (*Seq-1*) (Fig 7A), identified as the single strands of Duplex-1 by MALDI-TOF-MS through the detected masses: Seq-2, 2378.8 m/z and Seq-1, 2395.2 m/z (theoretical masses: Seq-2, 2379.6 m/z and Seq-1, 2394.6 m/z) (Fig 7C). This was in accord with previous reports that double-stranded oligonucleotides of this length denature under these HPLC conditions [46]. Duplex-1 was then incubated with **2** in a 4:1 ratio (**2**/DNA) for 24 hours at 25°C, and subjected to HPLC analysis. A new minor peak appeared at RT 19.30 min (Fig 7B) suggesting that an adduct had formed. Reaction was incomplete after 24 hours, with peaks corresponding to Seq-2 (RT 7.20 min; previously 7.16 mins) and Seq-1 (RT 7.52 min; previously 7.46 mins) remaining. Subsequent MALDI-TOF-MS analysis confirmed the presence of a 1:1 **2**/Seq-1 adduct with an observed mass of 2951.8 m/z (theoretical mass: 2951.21 m/z) (Fig 7C). It is noteworthy that in a similar manner to the denaturation that occurs during the HPLC process, under MALDI-TOF-MS conditions the duplex denatured into single strands with the PBD still attached to the guanine-containing strand. This observation was consistent with the data obtained for Hairpin-5, as Duplex-1 contains only one available guanine at its 5’-terminus. This further confirmed the ability of **2** to interact with a terminal guanine.

Next, Duplex-2 (S18 Fig) was studied. The resulting chromatogram provided two peaks at RT 7.13 min and RT 7.27 min corresponding to Seq-4 and Seq-3, respectively (S19 Fig). Their stoichiometry was confirmed by MALDI-TOF-MS with detected masses of m/z 2378.9 and 2394.5 (theoretical masses: Seq-4, 2379.6 m/z and Seq-3, 2394.6 m/z) (S19C Fig). After 24 hours incubation with **2** at 25°C in a 4:1 molar ratio (**2**/DNA), a new minor peak appeared at RT 19.30 min (S19B Fig). Reaction was incomplete after 24 hours (~14% adduct formation) with the two major peaks at RT 7.16 min (Seq-4) and RT 7.32 min (*Seq-3*) remaining (S19B Fig). The stoichiometry of the adduct formed was confirmed by MALDI-TOF-MS analysis as the 1:1 **2**/*Seq-3* adduct with a detected mass of 2952.0 m/z (theoretical mass: 2951.21 m/z) (S19C Fig). This result was similar to that obtained for Hairpin-6, and the slow extent of reaction may have been due to formation of the least preferred A-ring-5’ oriented adduct, as the guanine is close to the 3’-end of the duplex.

HPLC analysis of Duplex-3 (S18 Fig) provided two peaks at RT 6.86 min (*Seq-6*) and RT 7.40 min (*Seq-5*) (S20A Fig) identified by MALDI-TOF-MS as the component strands with observed masses of 2378.9 m/z (*Seq-5*) and 2394.2 m/z (*Seq-6*) (theoretical masses: *Seq-5*, 2379.6 m/z and *Seq-6*, 2394.6 m/z) (S20C Fig). Incubation for 24 hours at 25°C with **2** in a 4:1 ratio (**2**/DNA) provided a major new peak at RT 18.30 min, the stoichiometry of which was confirmed by MALDI-TOF-MS as the 1:1 **2**/Seq-6 adduct with an observed mass of 2951.5 m/z (theoretical mass: 2951.21 m/z) (S20C Fig). However, reaction was incomplete after 24 hours with ~43% adduct formation (S20B Fig). Interestingly, the extent of reaction of **2** with Duplex-3 was greater than that for Duplex-1 or Duplex-2, providing further evidence that a PBD can react at a terminal guanine base.

Lastly, HPLC analysis of Duplex-4 (S18 Fig) gave two peaks at RT 7.07 min (*Seq-8*) and RT 7.41 min (*Seq-7*) (S21A Fig) confirmed by MALDI-TOF-MS as *Seq-7* (observed mass, 2378.7 m/z; theoretical mass, 2379.6 m/z) and *Seq-8* (observed mass, 2394.9 m/z; theoretical mass, 2394.6 m/z) (S21C Fig). After incubation with **2** at 25°C in a 4:1 molar ratio (**2**/DNA), a new minor peak at RT 18.30 min was observed although reaction did not go to completion with only ~7% adduct formation (S21B Fig). The stoichiometry of the peak at RT 18.30 min was confirmed by MALDI-TOF-MS analysis as the 1:1 **2**/Seq-8 adduct with a detected mass of 2951.7 m/z (theoretical mass: 2951.21 m/z) (S21C Fig).

In summary, the results obtained from the interaction of **2** with Duplexes 1–4 (S18 Fig) were consistent with those obtained for Hairpins 5–8 (Fig 2). Furthermore, Duplex-1 and Duplex-3 both contain a guanine at the 5’-terminus, and **2** was able to react with both.

### Interaction of Anthramycin (1) and GWL-78 (3) with the Double-Stranded Inosine Modified AP-1 Sequence

Based on the data obtained for Hairpin-5, anthramycin (**1**) was not expected to react with Duplex-1 as it lacks a substituent at the C8-position that would help stabilize its accommodation in the DNA minor groove. In contrast, **3** was expected to react due to its C8-*bis*-pyrrole substituent. The results (S22A–S22C Fig) confirm that there is no reaction of **1** with Duplex-1 after 24 hours incubation. However, S22D–S22F Fig show that incubation of **3** with Duplex-1 for 24 hours provides an additional peak in the chromatogram at RT 14.27 min (S22E Fig), although reaction was incomplete with only ~8% adduct formation. The stoichiometry of the adduct formed was confirmed to be the 1:1 **3**/Seq-1 adduct by MALDI-TOF-MS analysis, with a detected mass of 2985.7 m/z (theoretical mass 2985.2 m/z) (S22F Fig).

### Molecular Dynamics Simulations

**Fig 8:**
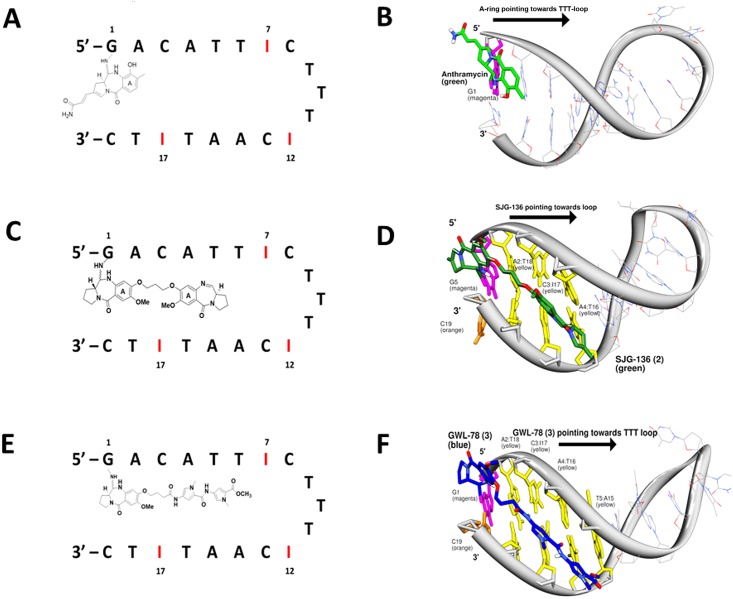
Schematic and molecular models of anthramycin (1), SJG-136 (2) and GWL-78 (3) covalently bound to the terminal guanine of Hairpin-5. **A**, Schematic model of **1** (green) covalently bound to G1 (magenta) of Hairpin-5 with the A-ring pointing toward the TTT-loop (A-ring-3’ orientation); **B**, Low energy snapshot of a 10 ns molecular dynamics simulation illustrating the C2-tail of **1** orienting outside of the DNA structure, suggesting that a DNA triplet is necessary for full accommodation of the molecule; **C**, Schematic model of **2** bound covalently to G1 of Hairpin-5, with the bulk of the molecule positioned within the minor groove (without cross-link formation) pointing toward the TTT-loop (*i*.*e*., the forward direction); **D**, Low energy snapshot of a molecular dynamics simulation (10 ns) of **2** covalently bound to the 5’-terminal G1 of Hairpin-5, illustrating the good accommodation of **2** (green sticks) within the minor groove and the non-covalent interactions between the central methylene linker of the ligand and the A2:T18 and C3:I17 base-pairs. **E**, Schematic model of **3** covalently bound to G1 of Hairpin-5, with the heterocyclic C8-sidechain pointing towards the TTT-loop; **F**, Low energy snapshot of a 10 ns molecular dynamics simulation of **3** (blue) covalently bound to G1 (magenta) of Hairpin-5, illustrating the comfortable accommodation of the C8-poly-pyrrole tail of **3** in the minor groove. It is likely that the poly-pyrrole tail forms non-covalent interactions with 5’-ACAT-3’ at 5’-positions 2–4 (yellow), guiding the PBD core to the G1 base.

Molecular dynamics simulations were carried out to try to predict the most preferred reacting guanine(s) in the AP-1 hairpin sequence (Fig 8). In these simulations, **2** was covalently bound to every potentially reacting guanine base (*i*.*e*., G1, G7, G12 and G17) in an effort to rationalize the structures of the adducts formed in the HPLC studies. As **2** is known to form mono-adducts and inter- and intrastrand cross-links [9, 33], all of these adduct types were investigated and, in the case of mono-adducts, both loop-facing (*i*.*e*., forward) and non-loop-facing (*i*.*e*., reverse) orientations were considered. The study design also considered potential reaction sites based on the span of the molecule. For example, in the case of G7 and G12, mono-adducts with the bulk of the molecule pointing toward the loop were not analyzed due to potential steric hindrance with the TTT-loop. However, adducts at G17 in both orientations were considered despite the fact that the significant bulk of a 3’-oriented adduct would be positioned outside of the minor groove environment.

Potential energy calculations (kcal/mol) on each adduct were undertaken to try to establish the most preferred reacting guanines for either mono-adduct or cross-link formation. Although **2** is known to prefer to form cross-linked adducts over a six or seven base-pair sequence (*i*.*e*., Pu-GATC-Py, Pu-GATG-Py, Pu-GAATG-Py or Pu-GAATC-Py [9]), initial potential energy calculations suggested that the shorter interstrand cross-linked adduct spanning 5’-GAC-3’ (*i*.*e*., G1-G17) is preferred by 12.41 kcal/mol (S27 Fig) compared to the extended G7-G17 interstrand cross-linked adduct (*i*.*e*., 5’-CATTG-3’) (Fig 3), despite the G1-bonded PBD unit interacting with two rather than the usual three base pairs at the terminal 5’-guanine. Although this result was surprising, especially as the extended 5’-ATT-3’ bases in the center of the hairpin should form van der Waals interactions with the C8/C8’-linker of **2**, it is possible that DNA breathing and base-pair separation affect the ability of **2** to stretch across the extended 5’-CATTG-3’ sequence which may have a detrimental effect on potential energy values. Furthermore, previous studies have suggested that the A-ring-3’ orientation for a PBD (*i*.*e*., 5’-GAC-3’) is preferred to A-ring-5’ (*i*.*e*., 5’- CATTG-3’), and although less favorable than Pu-GATC-Py, a 5’-GAC-3’ adduct of **2** has been previously reported [9].

For possible mono-adducts, the potential energy calculations suggested that G7 is the preferred reacting guanine (S23A Fig) in the case of SJG-136 (**2**). During simulations of this adduct, a stabilizing H-bond was observed to form between the N10-H of **2** and the N3 of A14 (S23B Fig). Furthermore, the second PBD unit of the dimer formed favourable non-covalent interactions with the central AT-tract of the sequence (*i*.*e*., 5’-ATT-3’), with both interactions likely to contribute to enhanced stability within the minor groove environment. The potential energy calculations suggested a hierarchy of mono-adduct formation of G7 > G17 (in the *forward* orientation) > G1 > G12 > G17 (in the *reverse* orientation). This ranking was broadly consistent with the observations from the inosine replacement experiments which suggested that G7 and G17 are favoured for covalent interaction based on the faster reaction rates of Hairpins 1, 2, 4, 6 and 8, and Duplexes 2 and 4. Conversely, potential energy calculations predicted that less favorable binding sites would include G12 where the TTT-loop is likely to inhibit interaction of **2**, or G17 (in the *reverse* orientation) in which case the second PBD unit of the dimer would be positioned outside of the groove where it would not be able to form non-covalent interactions with DNA bases in the minor groove, and would not be well-solvated due to its lipophilicity.

It is noteworthy that potential energy calculations for the bonding of **2** to G1, both in mono-adduct and cross-linking modes, suggest that it is viable as a reacting guanine. G1 adducts were observed by HPLC for the reaction of both **2** and **3** with 5’-GACATTIC-TTT-ICAATITC-3’ (Hairpin-5) where G1 is the only available guanine for reaction, and also in the sequence 5’-GATATTAT-TTT-ATAATATC-3’ (Hairpin-9) which is devoid of other reactive guanines. However, when anthramycin (**1**) was reacted with the same sequences, no adducts were formed. Although molecular dynamics simulations of **1** covalently bound to G1 suggested that an adduct in the C11-*S* configuration is theoretically possible, it is likely that the lack of non-covalent interactions from a C8-substituent, as available in the case of **2** and **3**, leads to low overall stabilization of the adduct. Further evidence for this was obtained through molecular dynamics simulations of **2** and **3** bound non-covalently to Hairpin-5, where non-covalent interactions from the C8-pyrrole chain (in the case of **3**) and the non-alkylating PBD unit (in the case of **2**), oriented the alkylating PBD component over G1 for covalent attack. However, in the case of **1** which does not have a C8-side chain, it moved up and down the minor groove during the simulation and failed to align over G1 to allow for attack by the C2-amino group.

Simulations of **2**, **3** and **1** covalently bound to G1 of 5’-GACATTIC-TTT-ICAATITC-3’ (Hairpin-5) were also undertaken with the ligands pointing away from the loop (S24, S25 and S26 Figs), to investigate the orientation of the G1 adducts. Potential energy calculations of **2** and **3** in both orientations suggested a potential energy difference of between 40 kcal/mol and 43 kcal/mol, respectively, in favour of the loop-facing orientation. This most likely reflected the unfavorable energetics when the bulk of the molecules point out of the DNA helix into a water environment rather than laying in the minor groove where they can provide adduct stabilization through non-bonding interactions. As anticipated, the energy difference between the two possible orientations of **1** covalently bonded to G1 was significantly less (*i*.*e*., 2.5 kcal/mol; -2736.10 kcal/mol *versus* -2733.60 kcal/mol) presumably due to the lack of a C8-substituent that can provide adduct stabilization through non-covalent interactions.

## Discussion

The results presented here initially demonstrated that PBDs such as **1**, **2** and **3** can covalently bind to the consensus DNA sequence for the oncogenic transcription factor AP-1. After this, inosine-modified AP-1 duplexes and hairpins were used to study the potential of the PBDs to form mono-alkylated and inter- and intrastrand cross-links at the various guanines within the duplex and hairpin sequences.

On the basis of results from previous studies [45], it was initially assumed that the adduct formed between SGJ-136 (**2**) and AP-1 (Hairpin-1) would be the G7/G17 interstrand cross-link (Fig 3), even though it is an extended interstrand cross-link (*i*.*e*., 3’-GTAAC-5’) compared to the most frequently reported interstrand cross-link, 5’-GATC-3’. The HPLC/MS experiments demonstrated that this was the case, and the extent of reaction of **2** with Hairpin-2 (where only G7 and G17 are available for covalent attack) after 24 hours was similar to that for Hairpin-1 (*i*.*e*., 100% adduct formation in 24 hours). Molecular modelling studies and potential energy calculations confirmed the feasibility of this extended adduct, although suggested that some distortion of the helix may result.

Other potential interstrand cross-links were then investigated using Hairpin-3 and Hairpin-4 (*i*.*e*., G1-G12 and G1-G17 cross-links, respectively). Although adducts formed with these hairpins, the extent of reaction was much lower than for the parent AP1 hairpin, suggesting that the G1-G12 and G1-G17 cross-linked adducts are less favourable than the G7-G17 cross-link.

Mono-adduct formation was investigated using Hairpins 5–8 and Duplexes 1–4 which each had only one reactive guanine available for covalent bonding of a PBD. From these experiments it became clear that SJG-136 (**2**) can covalently bond to a 5’-terminal guanine with one of its PBD units spanning only two base pairs. The most likely explanation for this, supported by the results of molecular modeling, is that one PBD unit of **2** bonds to the terminal 5’-guanine with its C-ring protruding just outside of the minor groove, and with the second PBD unit positioned in the minor groove (pointing toward the loop) stabilized by non-covalent interactions but not forming a second cross-linking covalent bond (Fig 8C). This hypothesis was supported by a molecular dynamics simulation (Fig 8D) which suggested that a stable adduct should result with **2** arranged in this way. The fact that adduct formation did not go to completion after 24 hours is also consistent with a mono-alkylation event in which the PBD is interacting with two rather than the usual three base pairs at the alkylation site. Modeling studies also suggested that the alternative arrangement, with one PBD unit of **2** bound to the terminal guanine but facing out of the minor groove would be unlikely due to the high energy associated with one PBD unit being positioned outside of the minor groove in the solvent environment.

Analogous experiments with the C8-*bis-*pyrrole-substituted PBD monomer GWL-78 (**3**) and the non-C8-substituted PBD monomer anthramycin (**1**) further supported the requirement for a C8-side chain to facilitate terminal-G bonding. For example, an adduct of Hairpin-5 was observed after reaction with **3**, but not with **1** (S17 Fig). In a similar manner to **2**, it is likely that the C8-sidechain of **3** facilitates formation of the adduct through non-covalent interactions (*i*.*e*., hydrogen bond formation, electrostatic forces and van der Waals interactions) with bases in the DNA minor groove. On the other hand, anthramycin (**1**) fails to react due to the lack of a C8-sidechain. These observations were fully supported by molecular dynamics simulations, where a greater stabilization of DNA was observed for **2** and **3**, compared to **1**.

As Hairpins 2–8 have two or three of their guanines replaced with inosine bases, we carried out control experiments to exclude the possibility that inosines may modify the conformation of the hairpin or duplex sequences and thus potentiate covalent interaction of PBDs with their G1 bases. However, Hairpin-9, which contains a 5’-terminal guanine but with inosine-cytosine base-pairs replaced with adenine-thymine base pairs, still reacted with SJG-136 (**2**) and GWL-78 (**3**) at approximately the same rate whereas the C8-unsubstituted PBD monomer anthramycin (**1**) failed to react, demonstrating that inosine substitution has no effect on reactivity. These observations were supported by molecular dynamics simulations which showed negligible differences in the propensity of Hairpin-5 or Hairpin-9 to form an adduct with PBDs **2** and **3**.

Taken together, these results suggest that C8-substitution of a PBD molecule can facilitate binding to a terminal guanine base. Conversely, anthramycin (**1**), which lacks a bulky minor-groove-interacting C8-substituent, is unable to interact with a guanine located at the 5’-terminus of a DNA hairpin. In particular, SJG-136 (**2**) can bond to the 5’-terminal guanine of Hairpin-2 and Duplex-1 because the second PBD unit joined through the C8-O-(CH_2_)_3_-O-C8’ linker can provide stabilization by interacting in the minor groove through hydrogen bond formation, electrostatic forces and van der Waals interactions, even if there is no suitable guanine available for cross-link formation.

## Conclusion

This is the first report of PBD molecules covalently bonding to a guanine base located at the end of a DNA hairpin or duplex sequence. To date, PBD units have been reported to span three DNA base pairs with a preference for a 5’-Pu-G-Pu-3’ motif, and with the C11-position of the PBD bonding to the central guanine through an aminal bond. In the case of C8/C8’-linked dimers, the molecules have been reported to span 6 or 7 base pairs. Apart from adding to knowledge of the variety of PBD/DNA adduct types possible, the results reported here highlight the importance of the C8-sidechain of PBDs in determining whether a particular type of adduct (in this case a terminal guanine adduct) can form. Finally, the observation of G-terminal adducts may have implications for the mechanism of cellular cytotoxicity and *in vivo* antitumour activity of C8-substituted PBD Monomers and C8-linked PBD Dimers. Until now it was thought that PBDs exert their biological activity by forming mono-alkylated or intra- or interstrand cross-linked adducts within stretches of DNA, thus blocking processes such as transcription factor binding, transcription or enzyme processing, or leading to stalling at the replication fork. However, the observation reported here suggests that a mechanism involving the binding of C8-subtituted PBDs to the ends of DNA double-strand breaks in cells may also be operative. This finding has particular significance for the mechanism of action of the PBD-based Antibody-Drug Conjugates currently in clinical evaluation and at the pre-clinical stage.

## Supporting Information

S1 Fig HPLC/MS data obtained from the interaction of SJG-136 (2) and Hairpin-1. **A**, HPLC chromatogram showing the annealed Hairpin-1 sequence alone at RT 7.31 min; **B**, HPLC chromatogram after incubation of the annealed Hairpin-1 sequence with SJG-136 (**2**) for 24 hours showing 100% conversion to an adduct at RT 7.53 min with complete loss of the original hairpin peak at RT 7.31 min; **C**, MALDI-TOF spectrum of the adduct at RT 7.53 min in Chromatogram **B** above. Observed mass of adduct: 6351.2 m/z (theoretical mass: 6350.41 m/z), observed mass of DNA Hairpin-1 alone from Chromatogram A: 5794.8 m/z (theoretical mass: 5793.8 m/z).
(TIF)

S2 Fig HPLC/MS data obtained from the interaction of SJG-136 (2) and Hairpin-2. **A**, HPLC chromatogram showing the annealed Hairpin-2 sequence alone at RT 6.78 min; **B**, HPLC chromatogram after incubation of annealed Hairpin-2 with **2** for 24 hours, showing 100% conversion to an adduct at RT 8.40 min with complete loss of the original hairpin peak at RT 6.78 min; **C**, MALDI-TOF spectrum of the adduct (from peak at RT 8.40 min in Chromatogram **B** above). Observed mass of 1:1 **2**/Hairpin-2 adduct: 6319.5 m/z (theoretical mass: 6320.4 m/z), observed mass of DNA Hairpin-2 alone from Chromatogram: 5764.5 m/z (theoretical mass: 5763.8 m/z).
(TIF)

S3 Fig HPLC/MS data obtained from the interaction of SJG-136 (2) and Hairpin-3. **A**, HPLC chromatogram of annealed Hairpin-3 at RT 7.43 min; **B**, Annealed Hairpin-3 after incubating with **2** for 24 hours, showing one new major adduct peak at RT 8.08 min with reaction not complete after 24 hours; **C**, MALDI-TOF spectrum of the adduct (from peak at RT 8.08 min in Chromatogram **B** above). Observed mass of Hairpin-3: 5764.2 m/z (theoretical mass: 5763.8 m/z); Observed mass of **2**/Hairpin-3 adduct: 6321.0 m/z (theoretical mass: 6320.4 m/z).
(TIF)

S4 Fig HPLC/MS data obtained from the interaction of SJG-136 (2) and Hairpin-4. **A**, HPLC chromatogram of annealed Hairpin-4 at RT 7.44 min; **B**, Annealed Hairpin-4 after incubating with **2** for 24 hours, showing one new major adduct peak at RT 8.04 min with reaction not complete after 24 hours; **C**, MALDI-TOF spectrum of the adduct (from peak at RT 8.04 min in Chromatogram **B** above). Observed mass of Hairpin-4: 5764.1 m/z (theoretical mass: 5763.8 m/z); Observed mass of **2**/Hairpin-4 adduct: 6321.9 m/z (theoretical mass: 6320.4 m/z).
(TIF)

S5 Fig HPLC/MS data obtained from the interaction of SJG-136 (2) and Hairpin-5. **A**, HPLC chromatogram of annealed Hairpin-6 at RT 8.01 min; **B**, Annealed Hairpin-6 after incubating with **2** for 24 hours, showing one new major adduct peak at RT 9.24 min with reaction not complete after 24 hours; **C**, MALDI-TOF spectrum of the adduct (from peak at RT 9.24 min in Chromatogram **B** above). Observed mass of Hairpin-6: 5749.2 m/z (theoretical mass: 5748.8 m/z); Observed mass of **2**/Hairpin-6 adduct: 6306.1 m/z (theoretical mass: 6305.41 m/z).
(TIF)

S6 Fig HPLC/MS data obtained from the interaction of SJG-136 (2) and Hairpin-6. **A**, HPLC chromatogram of annealed Hairpin-7 at RT 8.14 min; **B**, Annealed Hairpin-7 after incubating with **2** for 24 hours, showing one new major adduct peak at RT 9.07 min with reaction not complete after 24 hours; **C**, MALDI-TOF spectrum of the adduct (from peak at RT 9.07 min in Chromatogram **B** above). Observed mass of Hairpin-7: 5748.6 m/z (theoretical mass: 5748.8 m/z); Observed mass of **2**/Hairpin-7 adduct: 6305.0 m/z (theoretical mass: 6305.41 m/z).
(TIF)

S7 Fig HPLC/MS data obtained from the interaction of SJG-136 (2) and Hairpin-7. **A**, HPLC chromatogram of annealed Hairpin-8 at RT 8.03 min; **B**, Annealed Hairpin-8 after incubating with **2** for 24 hours, showing one new major adduct peak at RT 8.86 min with reaction not complete after 24 hours; **C**, MALDI-TOF spectrum of the adduct (from peak at RT 8.86 min in Chromatogram **B** above). Observed mass of Hairpin-8: 5748.9 m/z (theoretical mass: 5748.8 m/z); Observed mass of **2**/Hairpin-8 adduct: 6305.6 m/z (theoretical mass: 6305.41 m/z).
(TIF)

S8 Fig HPLC Chromatogram of Hairpin-5 from the MS-MS Analyses. Base peak chromatogram of Hairpin-5 injected onto the reversed-phase column showing a peak eluting at RT 7.1 min.
(TIF)

S9 Fig ESI-MS spectrum of Hairpin-5 from the MS-MS Analyses. ESI mass spectrum of the chromatographic peak at 7.1 min for Hairpin-5 from S8 Fig at charge states (*m/z* 1915 [M-3H]^3-^ and 2873 [M-2H]^2-^).
(TIF)

S10 Fig ESI-MS spectrum of Hairpin-5 from the MS-MS Analyses. ESI mass spectrum of the chromatographic peak at 7.1 min for Hairpin-5 from S8 Fig at the expanded mass range of *m/z* 1910 to 1919.
(TIF)

S11 Fig MS/MS spectrum of Hairpin-5 from the MS-MS Analyses. MS/MS spectrum for *m/z* 1913 corresponding to the chromatographic peak at 7.1 min for Hairpin-5 from S8 Fig at collision energy 20.
(TIF)

S12 Fig MS/MS spectrum of Hairpin-5 from the MS-MS Analyses. MS/MS spectrum for *m/z* 1913 corresponding to the chromatographic peak at 7.1 min for Hairpin-5 from S8 Fig at collision energy 35.
(TIF)

S13 Fig Full ESI mass spectrum of Hairpin 5 from the MS-MS Analyses. Full ESI mass spectrum for the chromatographic peak eluting at 7.1 min for Hairpin-5 from S8 Fig at collision energy 40. The signal at *m/z* 1915 corresponds to the triply charged [M-3H]^3-^ ion of Hairpin-5.
(TIF)

S14 Fig MS/MS spectrum of Hairpin-5 from the MS-MS Analyses. MS/MS spectrum for *m/z* 1913 corresponding to the chromatographic peak at 7.1 min for Hairpin-5 from S8 Fig at collision energy 40.
(TIF)

S15 Fig Reconstructed ion chromatogram of the SJG-136 (2)/Hairpin-5 adduct from the MS-MS Analyses. Reconstructed ion chromatogram of *m/z* 1914 from SJG-136/DNA adduct formation at a molar ratio of 4:1 after 12h incubation, with Hairpin-5 eluting at 7.1 min and the SJG-136/DNA adduct eluting at 9.1 min.
(TIF)

S16 Fig ESI-MS spectrum of the SJG-136 (2)/Hairpin-5 adduct from the MS-MS Analyses. Full ESI mass spectrum for the chromatographic peak at 9.1 min (see S15 Fig) expanded in the mass range from *m/z* 2090 to 2106. The signal at *m/z* 2099.72 corresponds to the triply charged [M-3H]^3-^ ion of SJG-136/Hairpin-5.
(TIF)

S17 Fig HPLC/MS data obtained from the interaction of anthramycin (1) and GWL-78 (3) with Hairpin-5. **A**, HPLC chromatogram showing annealed Hairpin-5 at RT 7.90 min; **B**, Annealed Hairpin-5 after incubating with **1** for 24 hours showing no adduct formation after 24 hours; **C**, MALDI-TOF spectrum of **1** with Hairpin-5 confirming that no adduct had formed (Hairpin-5 observed mass: 5748.0 m/z, theoretical mass: 5748.8 m/z); **D**, Annealed Hairpin-5 at RT 8.69 min; **E**, Annealed Hairpin-5 after incubating with **3** for 24 hours showing the appearance of an adduct peak at RT 11.13 min with approximately 23% complete reaction after 24 hours; **F**, MALDI-TOF spectrum of **3**/Hairpin-5 confirming the identity of adduct formation (Hairpin-5 observed mass: 5749.5 m/z, theoretical mass: 5748.8 m/z, Hairpin-5 adduct observed mass: 6338.6 m/z, theoretical mass: 6339.4 m/z). The observed differences in the RT of Hairpin-5 alone (*i*.*e*., S17A Fig and S17D Fig) occurred due to mobile phase changes.
(TIF)

S18 Fig Structures of the inosine-modified DNA duplexes used in the study. Duplexes 1–4 are analogous to Hairpins 5–8 (Fig 2 of main paper), in that three of the four guanines have been replaced with inosines. The duplexes are 8 base pairs in length.
(TIF)

S19 Fig HPLC/MS data obtained from the interaction of SJG-136 (2) and Duplex-2. **A**, HPLC chromatogram of annealed Duplex-2 at RT 7.13 min (Seq-4) and RT 7.27 min (Seq-3); **B**, Annealed Duplex-2 after incubating with **2** for 24 hours showing a new adduct peak at RT 19.30 min (**2**/Seq-3), with the reaction not complete after 24 hours; **C**, MALDI-TOF spectrum of **2** with Duplex-2, confirming the stoichiometry of the adduct formed; single strands of Duplex-2, observed masses: Seq-4, 2378.9 m/z and Seq-3, 2394.5 m/z (theoretical mass: Seq-4 2379.6 m/z and Seq-3 2394.6 m/z), and Duplex-2 observed mass: 4773.9 m/z (theoretical mass: 4774.2 m/z), **2**/Seq-3 adduct observed mass: 2952.0 m/z (theoretical mass: 2951.21 m/z).
(TIF)

S20 Fig HPLC/MS data obtained from the interaction of SJG-136 (2) and Duplex-3. **A**, HPLC chromatogram of annealed Duplex-3 at RT 6.86 min (Seq-6) and RT 7.40 min (Seq-5); **B**, Annealed Duplex-3 after incubating with **2** for 24 hours showing a new adduct peak at RT 18.30 min (**2**/Seq-6), with the reaction not complete after 24 hours; **C**, MALDI-TOF spectrum of **2** with Duplex-3, confirming the stoichiometry of the adduct formed; single strands of Duplex-3, observed masses: Seq-5, 2378.9 m/z and Seq-6, 2394.2 m/z (theoretical mass: Seq-5 2379.6 m/z and Seq-6 2394.6 m/z), and Duplex-3 observed mass: 4774.6 m/z (theoretical mass: 4774.2 m/z), **2**/Seq-6 adduct observed mass: 2951.5 m/z (theoretical mass: 2951.21 m/z).
(TIF)

S21 Fig HPLC/MS data obtained from the interaction of SJG-136 (2) and Duplex-4. **A**, HPLC chromatogram of annealed Duplex-4 at RT 7.07 min (Seq-8) and RT 7.4 min (Seq-7); **B**, Annealed Duplex-4 after incubating with **2** for 24 hours showing a new adduct peak at RT 18.30 min (**2**/Seq-8), with the reaction not complete after 24 hours; **C**, MALDI-TOF spectrum of **2** with Duplex-4, confirming the stoichiometry of the adduct formed; single strands of Duplex-4, observed masses: Seq-7, 2378.7 m/z and Seq-8, 2394.9 m/z (theoretical mass: Seq-7 2379.6 m/z and Seq-8 2394.6 m/z), and Duplex-4 observed mass: 4774.8 m/z (theoretical mass: 4774.2 m/z), **2**/Seq-8 adduct observed mass: 2951.7 m/z (theoretical mass: 2951.21 m/z).
(TIF)

S22 Fig HPLC/MS data obtained from the interaction of GWL-78 (3) and anthramycin (1) with Duplex-1. **A**, HPLC chromatogram of annealed Duplex-1 at RT 6.39 min (Seq-2) and RT 6.76 min (Seq-1); **B**, After incubation with **1** for 24 hours showing no reaction; **C**, MALDI-TOF spectrum of **1** with Duplex-1 confirming no reaction; Single strands of Duplex-1 observed masses: Seq-2, 2378.9 m/z and Seq-1, 2394.8 m/z (theoretical masses: Seq-2, 2379.6 m/z and Seq-1, 2394.6 m/z); **D**, Annealed Duplex-1 at RT 6.39 min (Seq-2) and 6.76 min (Seq-1); **E**, Annealed Duplex-1 after incubating with **3** for 24 hours showing a new peak at RT 14.27 min; **F**, MALDI-TOF spectrum of **3** with Duplex-1 confirming the stoichiometry as the 1:1 **3**/Seq-1 adduct; Single strands of Duplex-1 observed masses: Seq-2, 2378.7 m/z and Seq-1, 2394.0 m/z (theoretical masses: Seq-2, 2379.6 m/z and Seq-1, 2394.6 m/z) and double-stranded Duplex-1 observed mass of 4774.3 m/z (theoretical mass: 4774.2 m/z), **3**/Seq-1 adduct observed mass of 2985.7 m/z (theoretical mass: 2985.2 m/z).
(TIF)

S23 Fig Schematic and molecular models of SJG-136 (2) covalently bound to Hairpin-1. Schematic and low energy snapshot of a molecular dynamics simulation showing the accommodation of **2** (green sticks) in the minor groove while covalently bonded to G7 of Hairpin-1 (see Fig 2 of main paper). The NH group of the PBD formed a H-bond with the adjacent A14 adenine base (yellow), and the molecule maintained good isohelicity with the minor groove for the duration of the simulation (10 ns).
(TIF)

S24 Fig Schematic and molecular models of SJG-136 (2) covalently bound to Hairpin-5. **A**, Schematic model of **2** (green) covalently bound to G1 (magenta) of Hairpin-5 with the A-ring pointing away from the TTT-loop (A-ring-3’ orientation), and the second PBD of the dimer orienting outside of the minor groove; **B**, Low energy snapshot of 10 ns molecular dynamics simulation illustrating the second PBD of **2** orienting outside of the DNA minor groove.
(TIF)

S25 Fig Schematic and molecular models of GWL-78 (3) covalently bound to Hairpin-5. **A**, Schematic model of **3** (blue) covalently bound to G1 (magenta) of Hairpin-5 with the A-ring pointing away from the TTT-loop (A-ring-3’ orientation), and the C8-poly-pyrrole chain orienting outside of the minor groove; **B**, Low energy snapshot of a 10 ns molecular dynamics simulation illustrating the C8-poly-pyrrole chain of **3** orienting outside of the DNA minor groove and forming stacking interactions with the G1 base.
(TIF)

S26 Fig Schematic and molecular models of anthramycin (1) covalently bound to Hairpin-5. **A**, Schematic model of **1** (light green) covalently bound to G1 (magenta) of Hairpin-5 with the A-ring pointing away from the TTT-loop (A-ring-3’ orientation); **B**, Low energy snapshot of a 10 ns molecular dynamics simulation illustrating anthramycin (**1**) potentially bound to the G1 base through interactions with a doublet rather than a traditional triplet.
(TIF)

S27 Fig Table of potential energy (kcal/mol) calculations determined over both 10ns and 50ns implicit solvent simulations. Calculations were undertaken with SJG-136 (**2**) covalently bound to every potential reacting guanine in the AP-1 consensus sequence. The order of preference of binding was identical in both 10ns and 50ns simulations.
(TIF)
