# Supplementary figures and images for: Covalent Bonding of Pyrrolobenzodiazepines (PBDs) to Terminal Guanine Residues within Duplex and Hairpin DNA Fragments
Source: PLoS One. 2016 Apr 7;11(4):e0152303. doi: 10.1371/journal.pone.0152303 (PMC4824457; doi:10.1371/journal.pone.0152303)

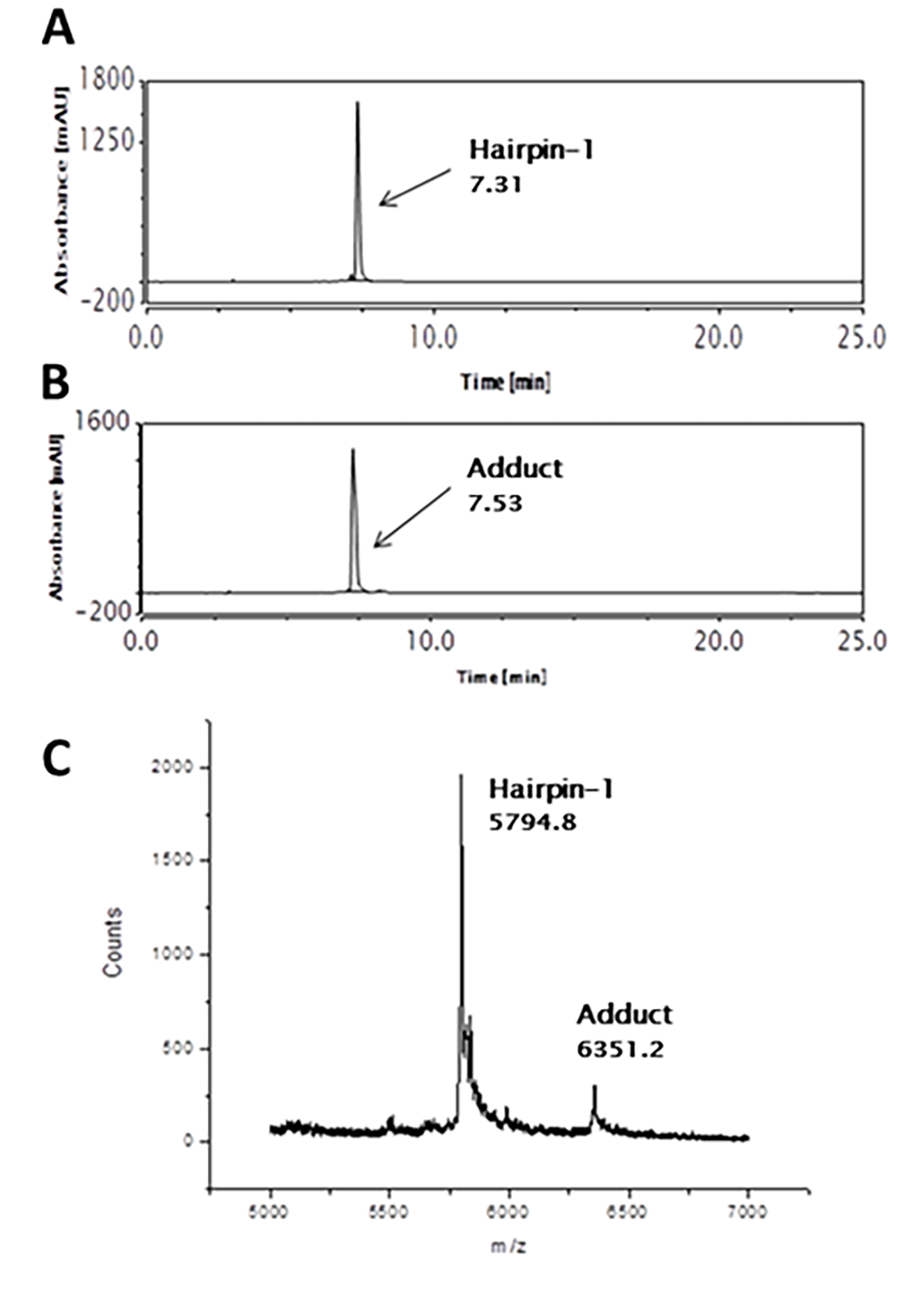

Supplement: S1 Fig — A, HPLC chromatogram showing the annealed Hairpin-1 sequence alone at RT 7.31 min; B, HPLC chromatogram after incubation of the annealed Hairpin-1 sequence with SJG-136 (2) for 24 hours showing 100% conversion to an adduct at RT 7.53 min with complete loss of the original hairpin peak at RT 7.31 min; C, MALDI-TOF spectrum of the adduct at RT 7.53 min in Chromatogram B above. Observed mass of adduct: 6351.2 m/z (theoretical mass: 6350.41 m/z), observed mass of DNA Hairpin-1 alone from Chromatogram A: 5794.8 m/z (theoretical mass: 5793.8 m/z). (TIF) [file pone.0152303.s001.tif]

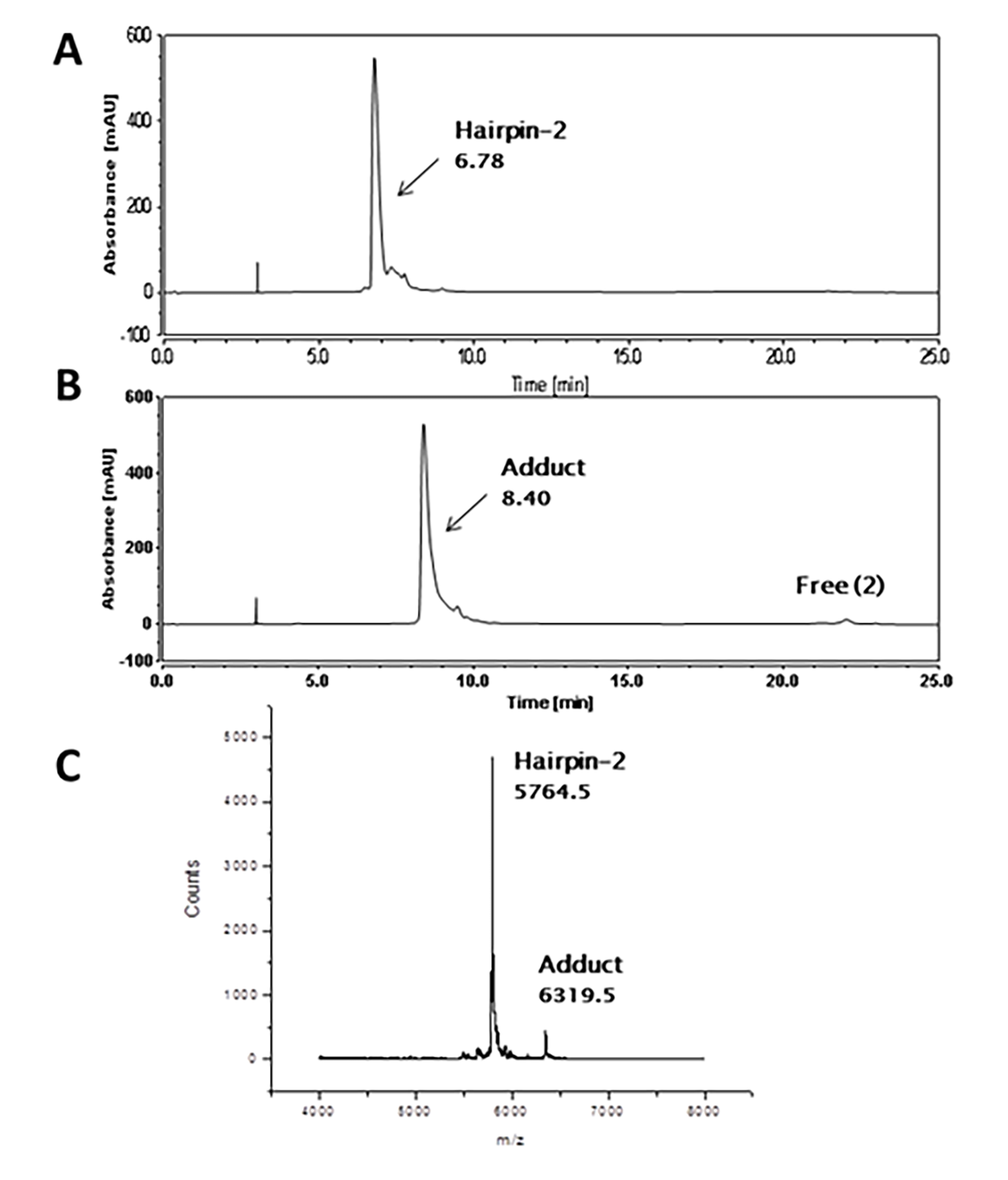

Supplement: S2 Fig — A, HPLC chromatogram showing the annealed Hairpin-2 sequence alone at RT 6.78 min; B, HPLC chromatogram after incubation of annealed Hairpin-2 with 2 for 24 hours, showing 100% conversion to an adduct at RT 8.40 min with complete loss of the original hairpin peak at RT 6.78 min; C, MALDI-TOF spectrum of the adduct (from peak at RT 8.40 min in Chromatogram B above). Observed mass of 1:1 2/Hairpin-2 adduct: 6319.5 m/z (theoretical mass: 6320.4 m/z), observed mass of DNA Hairpin-2 alone from Chromatogram: 5764.5 m/z (theoretical mass: 5763.8 m/z). (TIF) [file pone.0152303.s002.tif]

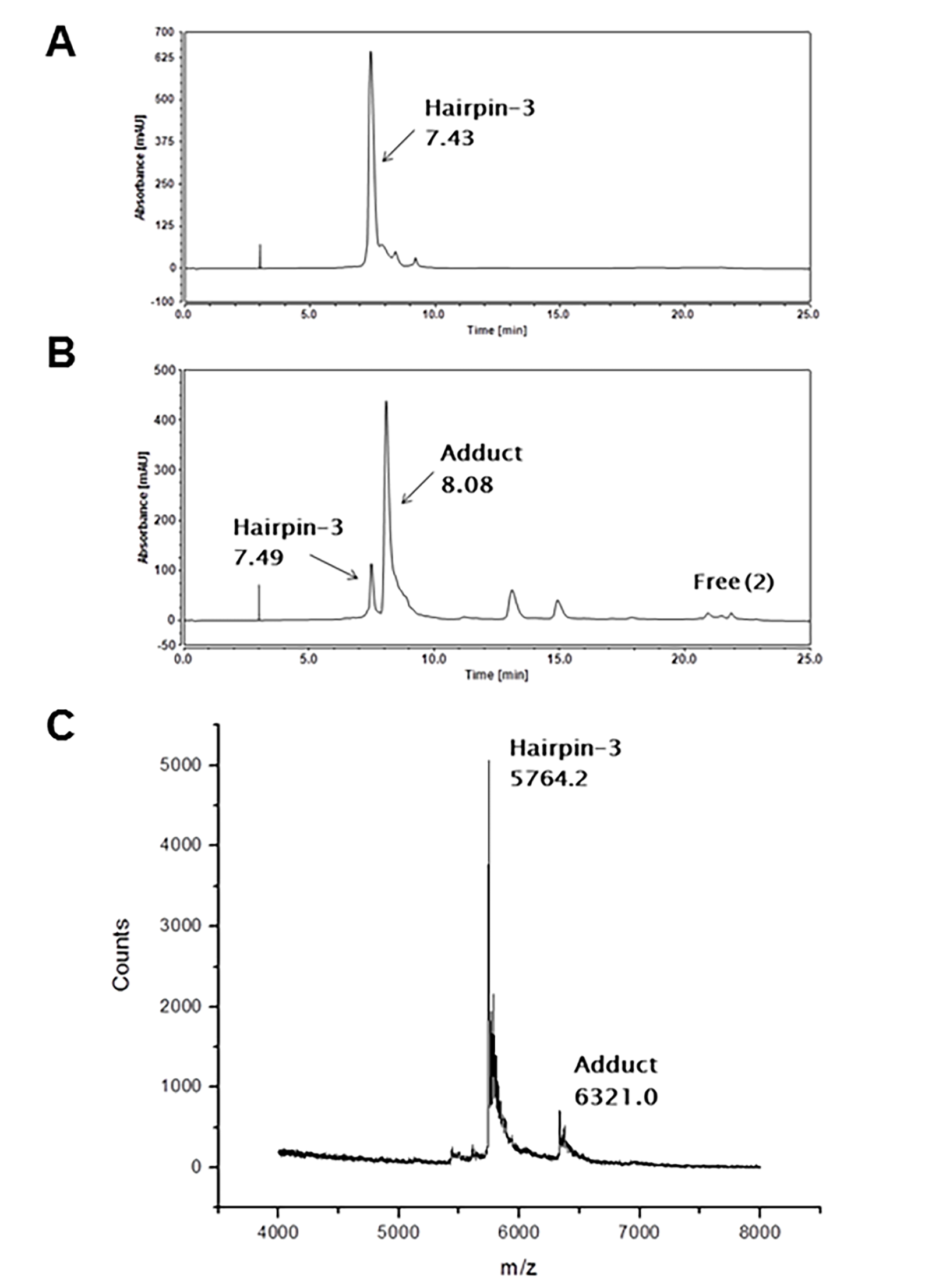

Supplement: S3 Fig — A, HPLC chromatogram of annealed Hairpin-3 at RT 7.43 min; B, Annealed Hairpin-3 after incubating with 2 for 24 hours, showing one new major adduct peak at RT 8.08 min with reaction not complete after 24 hours; C, MALDI-TOF spectrum of the adduct (from peak at RT 8.08 min in Chromatogram B above). Observed mass of Hairpin-3: 5764.2 m/z (theoretical mass: 5763.8 m/z); Observed mass of 2/Hairpin-3 adduct: 6321.0 m/z (theoretical mass: 6320.4 m/z). (TIF) [file pone.0152303.s003.tif]

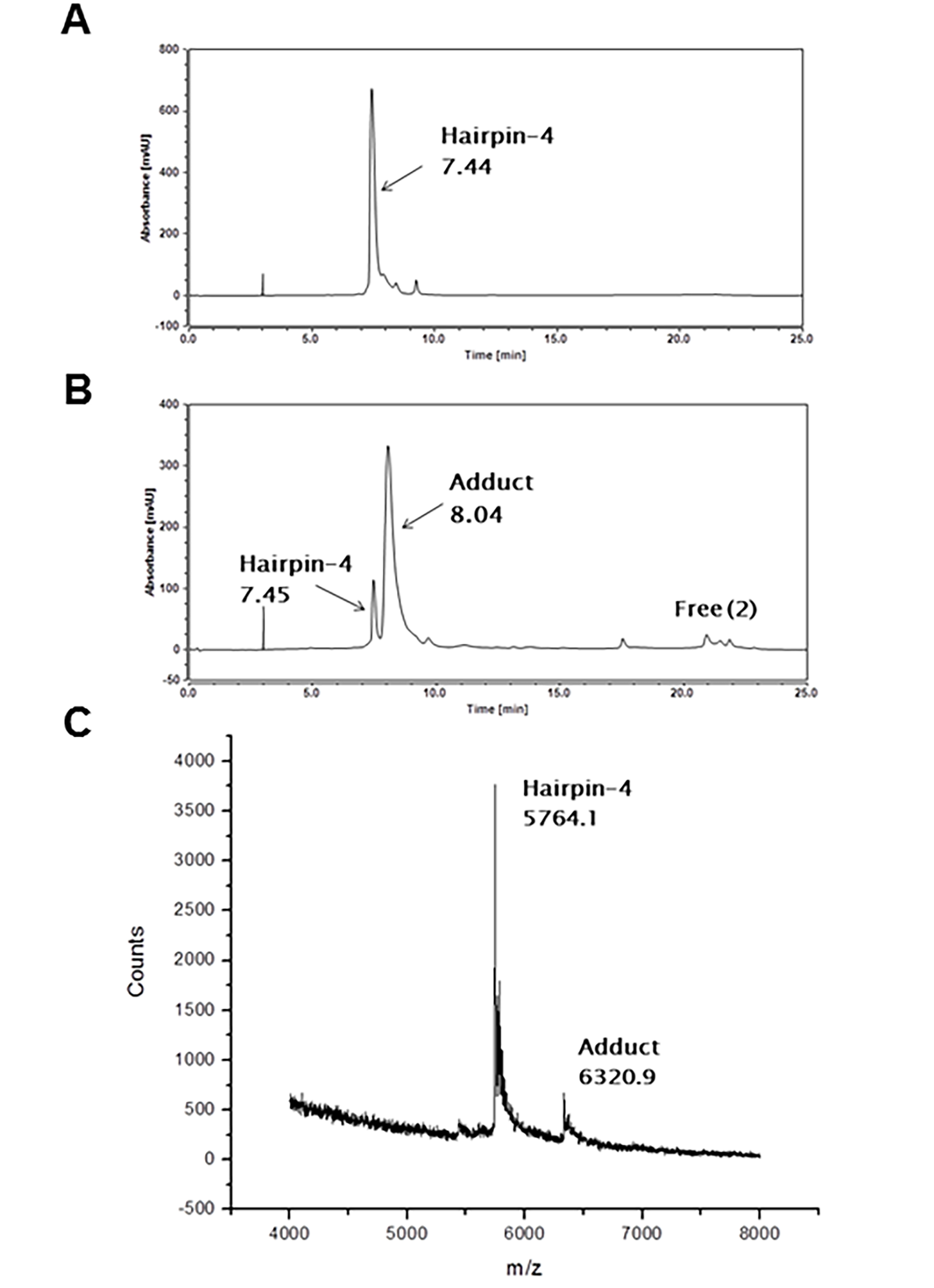

Supplement: S4 Fig — A, HPLC chromatogram of annealed Hairpin-4 at RT 7.44 min; B, Annealed Hairpin-4 after incubating with 2 for 24 hours, showing one new major adduct peak at RT 8.04 min with reaction not complete after 24 hours; C, MALDI-TOF spectrum of the adduct (from peak at RT 8.04 min in Chromatogram B above). Observed mass of Hairpin-4: 5764.1 m/z (theoretical mass: 5763.8 m/z); Observed mass of 2/Hairpin-4 adduct: 6321.9 m/z (theoretical mass: 6320.4 m/z). (TIF) [file pone.0152303.s004.tif]

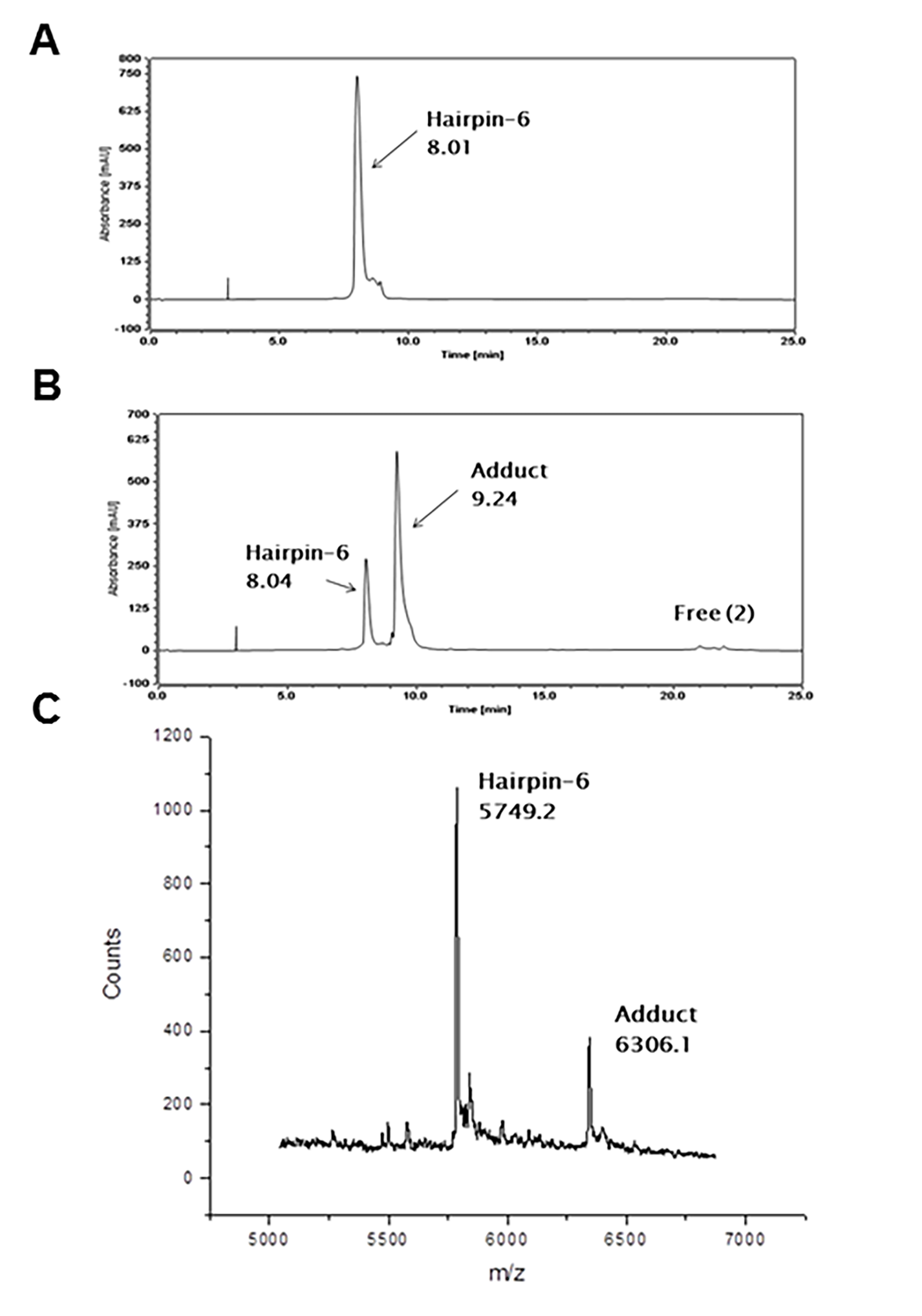

Supplement: S5 Fig — A, HPLC chromatogram of annealed Hairpin-6 at RT 8.01 min; B, Annealed Hairpin-6 after incubating with 2 for 24 hours, showing one new major adduct peak at RT 9.24 min with reaction not complete after 24 hours; C, MALDI-TOF spectrum of the adduct (from peak at RT 9.24 min in Chromatogram B above). Observed mass of Hairpin-6: 5749.2 m/z (theoretical mass: 5748.8 m/z); Observed mass of 2/Hairpin-6 adduct: 6306.1 m/z (theoretical mass: 6305.41 m/z). (TIF) [file pone.0152303.s005.tif]

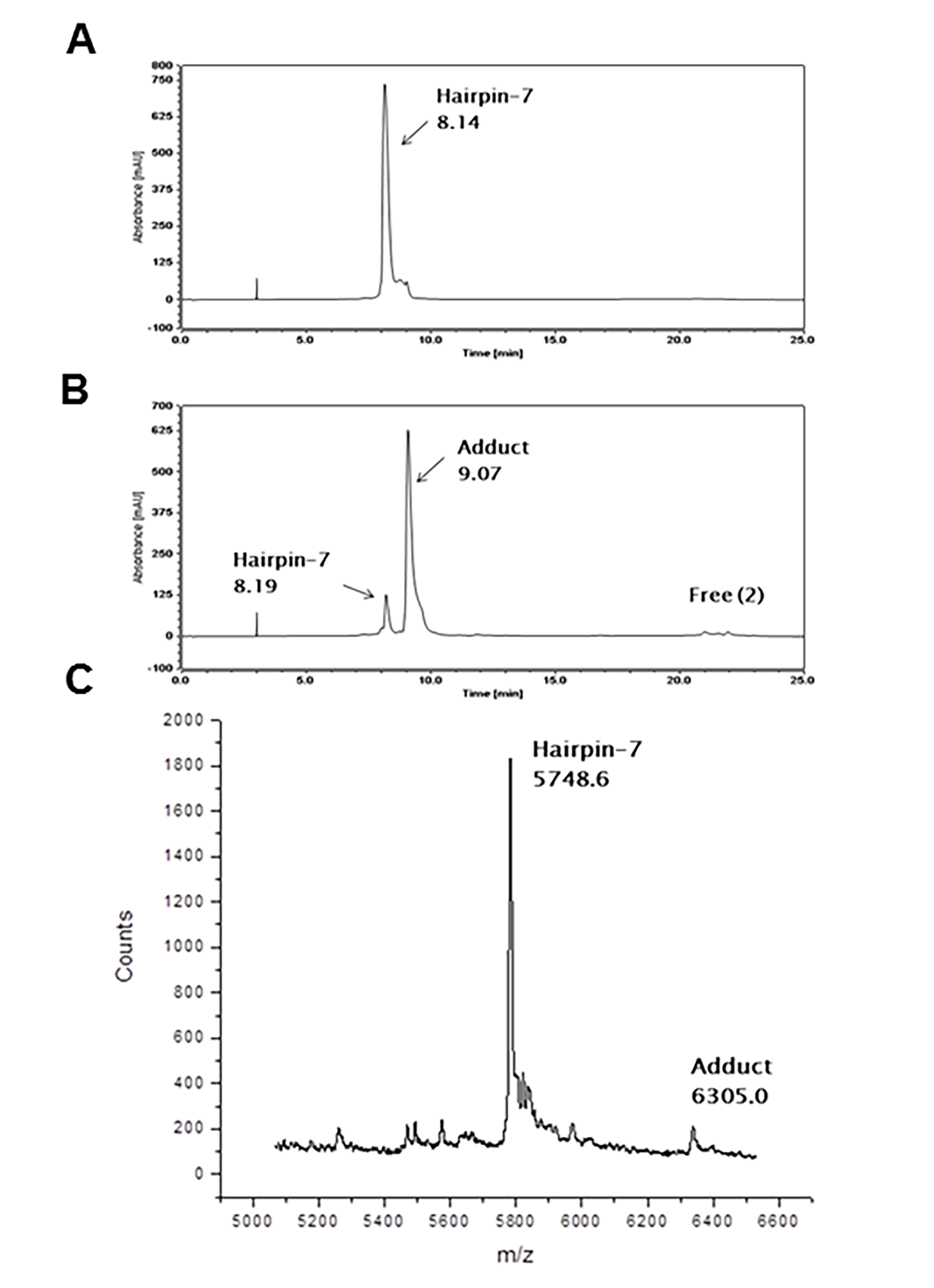

Supplement: S6 Fig — A, HPLC chromatogram of annealed Hairpin-7 at RT 8.14 min; B, Annealed Hairpin-7 after incubating with 2 for 24 hours, showing one new major adduct peak at RT 9.07 min with reaction not complete after 24 hours; C, MALDI-TOF spectrum of the adduct (from peak at RT 9.07 min in Chromatogram B above). Observed mass of Hairpin-7: 5748.6 m/z (theoretical mass: 5748.8 m/z); Observed mass of 2/Hairpin-7 adduct: 6305.0 m/z (theoretical mass: 6305.41 m/z). (TIF) [file pone.0152303.s006.tif]

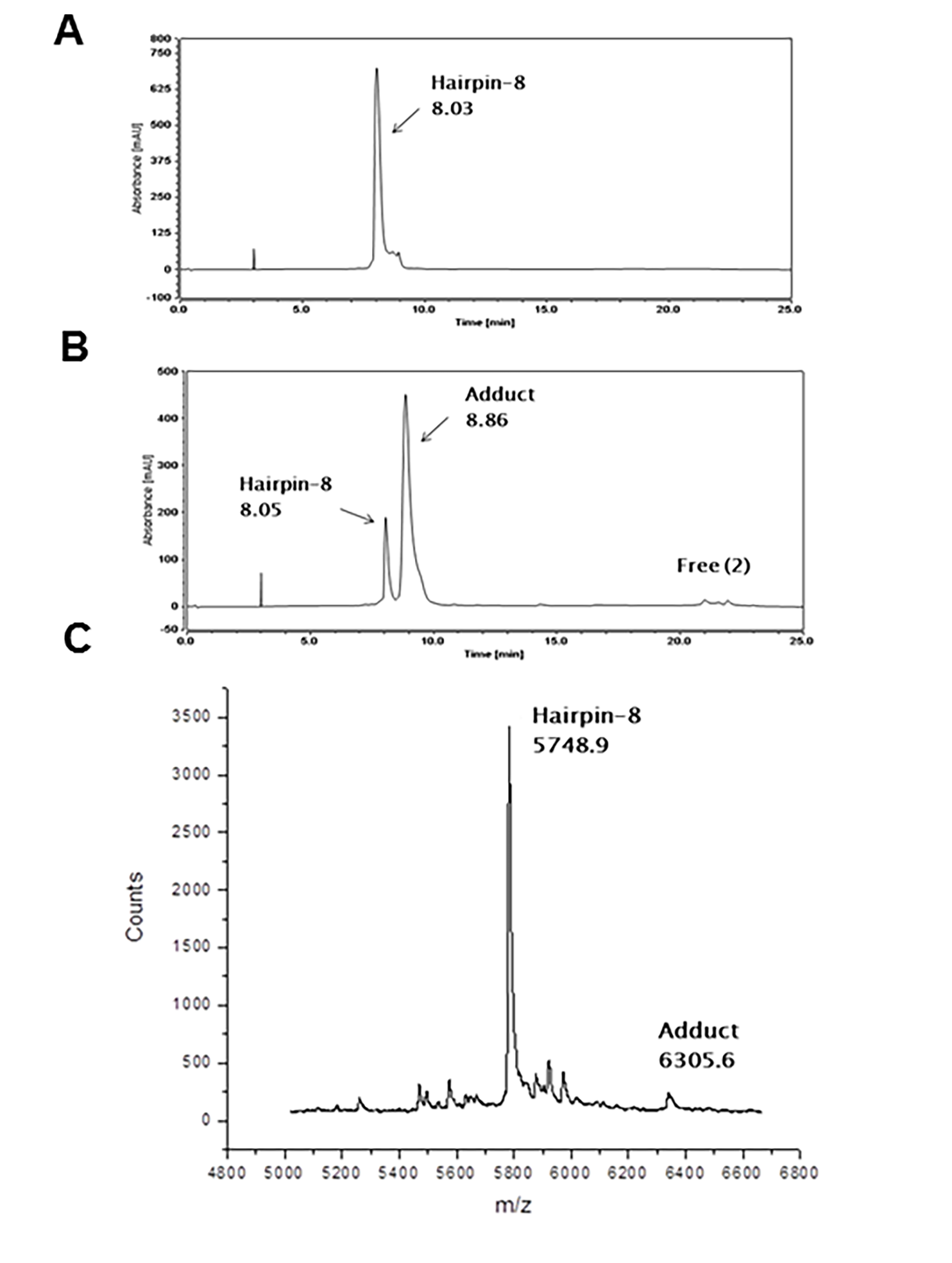

Supplement: S7 Fig — A, HPLC chromatogram of annealed Hairpin-8 at RT 8.03 min; B, Annealed Hairpin-8 after incubating with 2 for 24 hours, showing one new major adduct peak at RT 8.86 min with reaction not complete after 24 hours; C, MALDI-TOF spectrum of the adduct (from peak at RT 8.86 min in Chromatogram B above). Observed mass of Hairpin-8: 5748.9 m/z (theoretical mass: 5748.8 m/z); Observed mass of 2/Hairpin-8 adduct: 6305.6 m/z (theoretical mass: 6305.41 m/z). (TIF) [file pone.0152303.s007.tif]

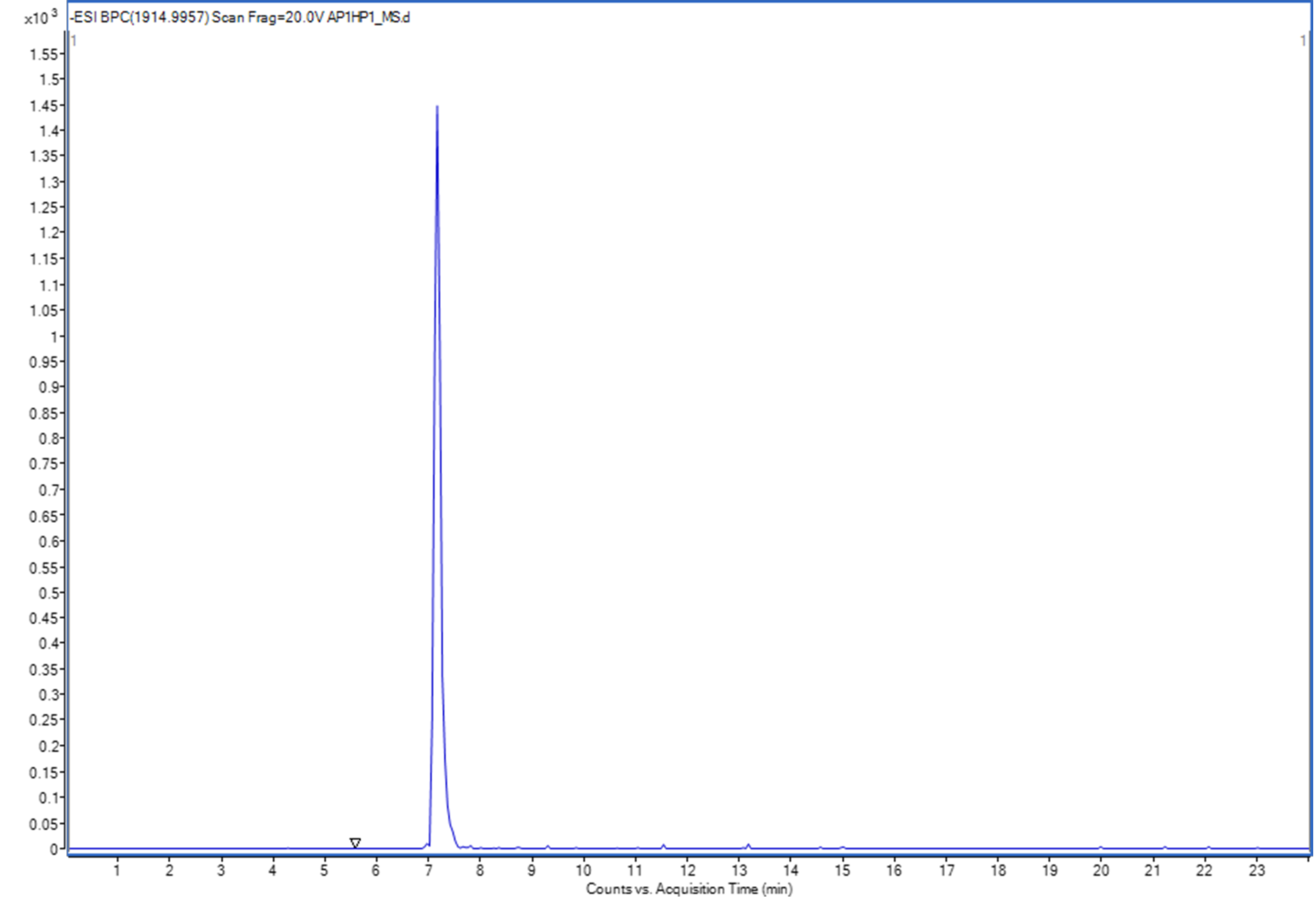

Supplement: S8 Fig — Base peak chromatogram of Hairpin-5 injected onto the reversed-phase column showing a peak eluting at RT 7.1 min. (TIF) [file pone.0152303.s008.tif]

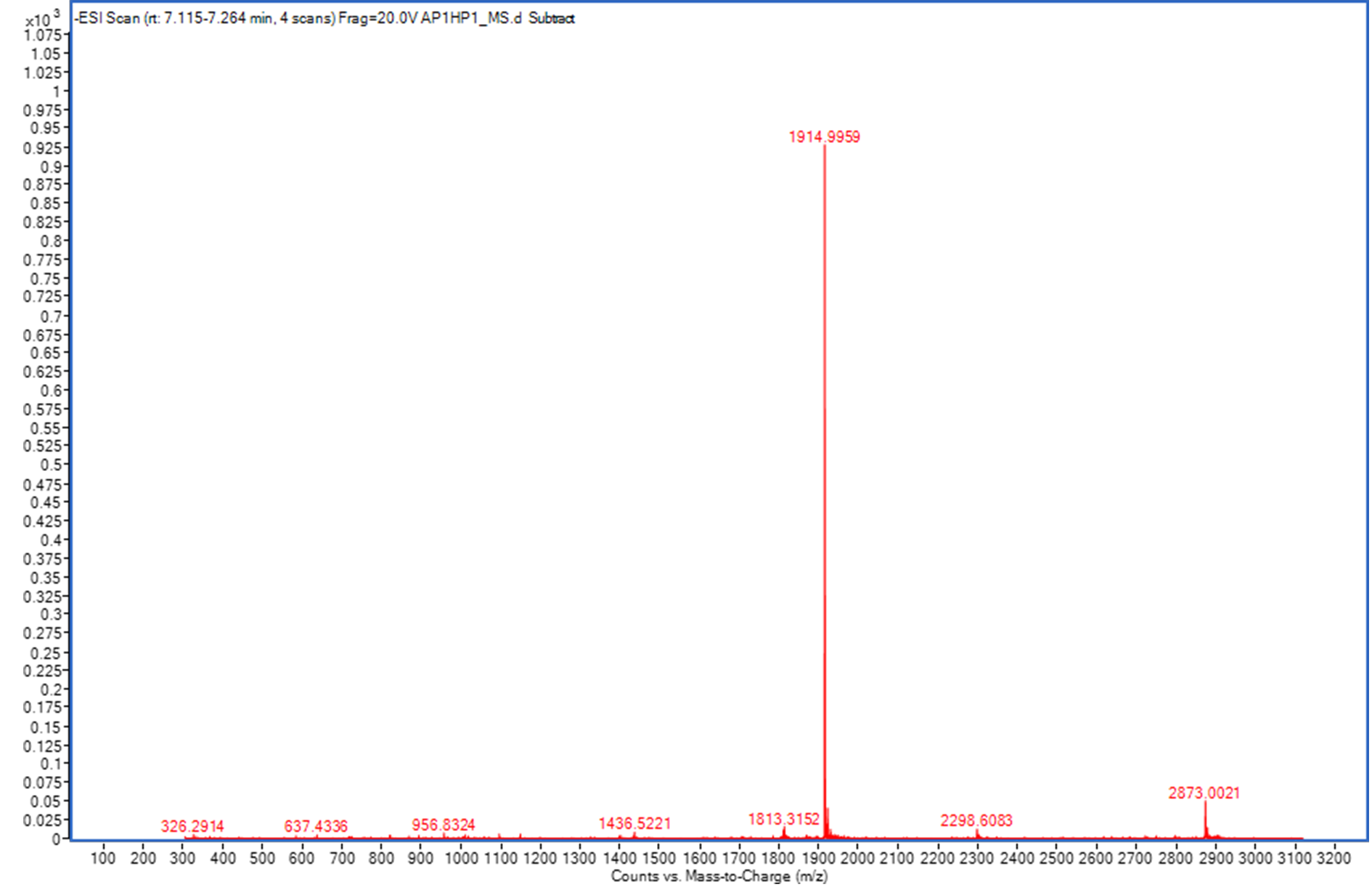

Supplement: S9 Fig — ESI mass spectrum of the chromatographic peak at 7.1 min for Hairpin-5 from S8 Fig at charge states (m/z 1915 [M-3H]3- and 2873 [M-2H]2-). (TIF) [file pone.0152303.s009.tif]

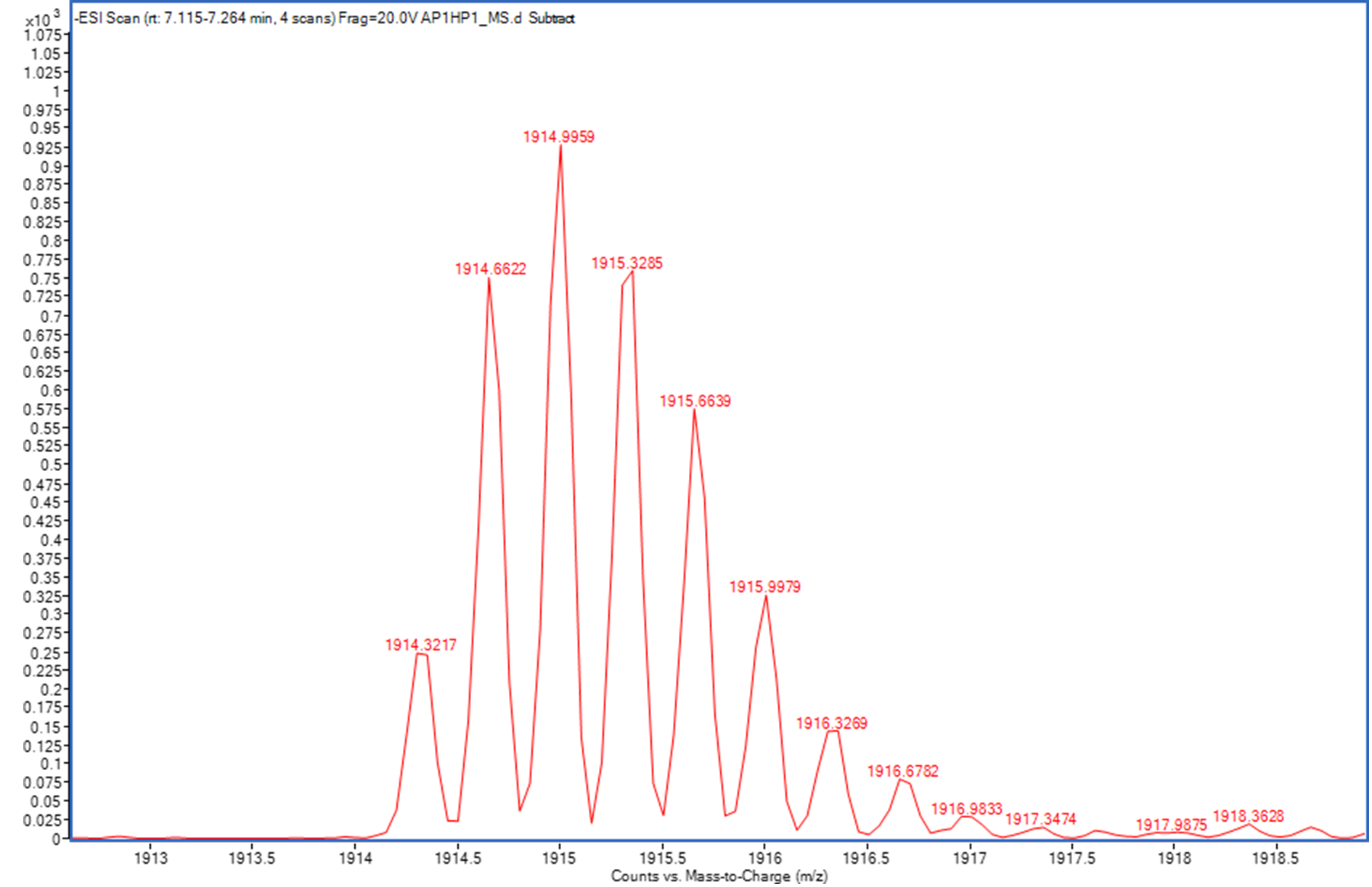

Supplement: S10 Fig — ESI mass spectrum of the chromatographic peak at 7.1 min for Hairpin-5 from S8 Fig at the expanded mass range of m/z 1910 to 1919. (TIF) [file pone.0152303.s010.tif]

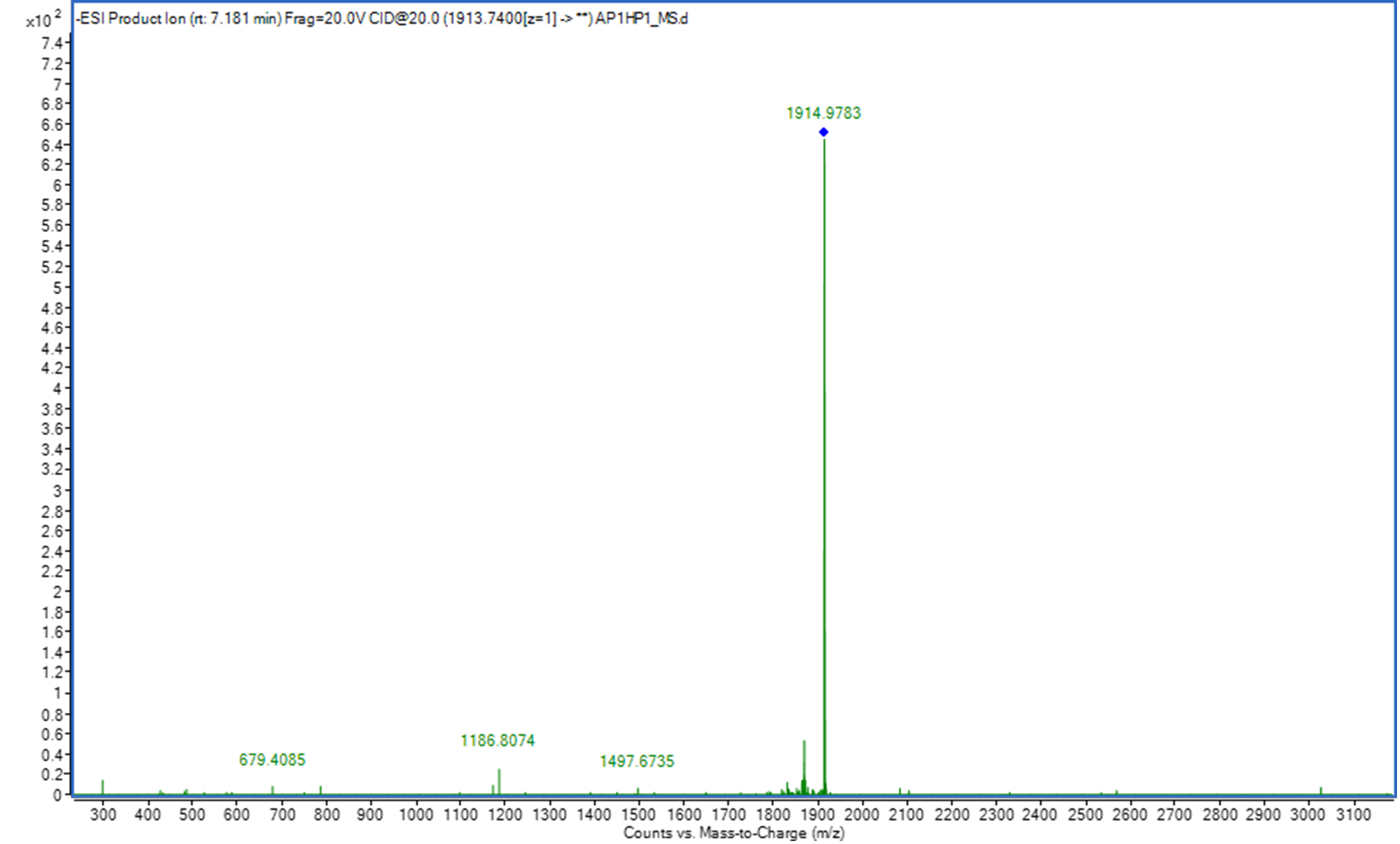

Supplement: S11 Fig — MS/MS spectrum for m/z 1913 corresponding to the chromatographic peak at 7.1 min for Hairpin-5 from S8 Fig at collision energy 20. (TIF) [file pone.0152303.s011.tif]

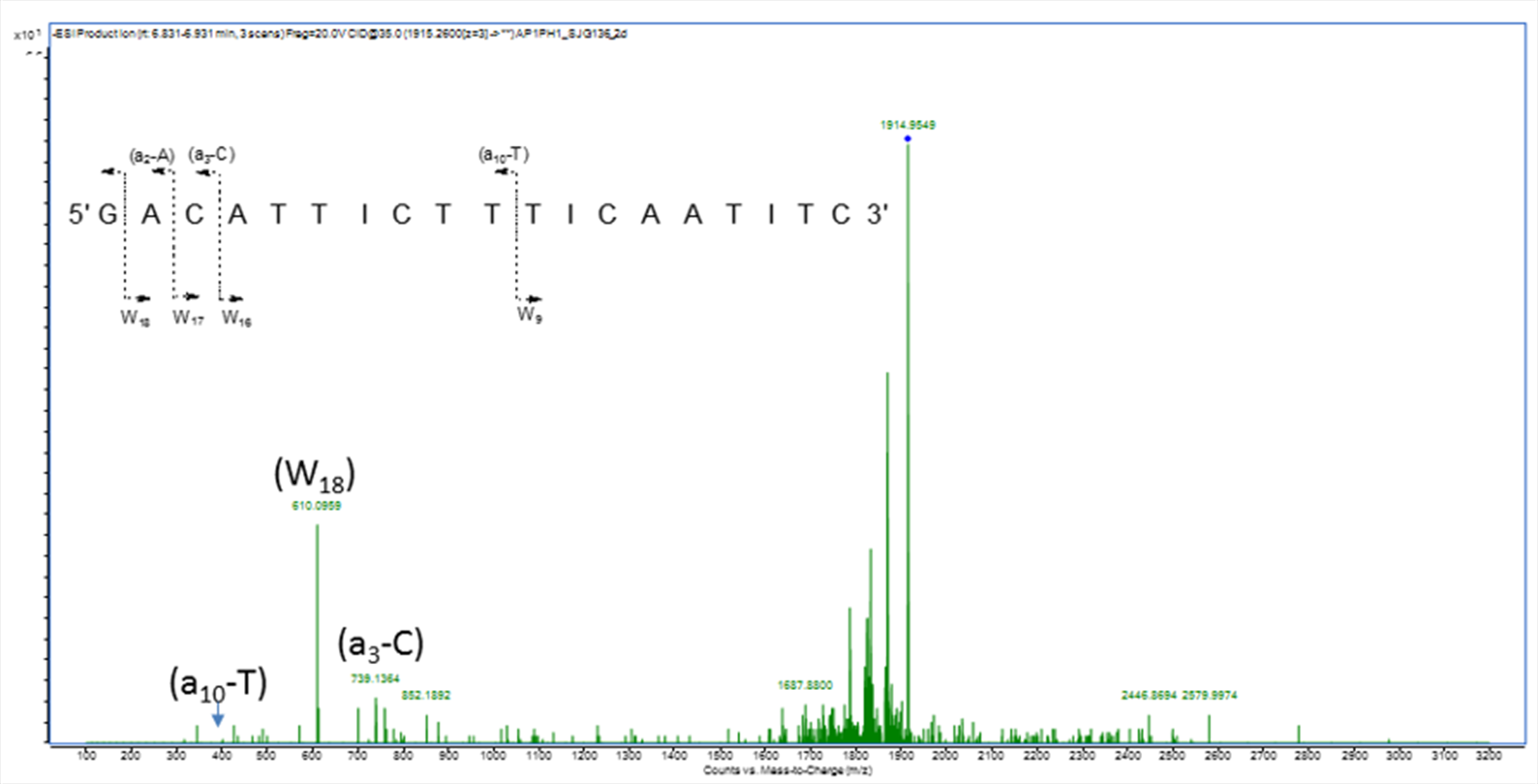

Supplement: S12 Fig — MS/MS spectrum for m/z 1913 corresponding to the chromatographic peak at 7.1 min for Hairpin-5 from S8 Fig at collision energy 35. (TIF) [file pone.0152303.s012.tif]

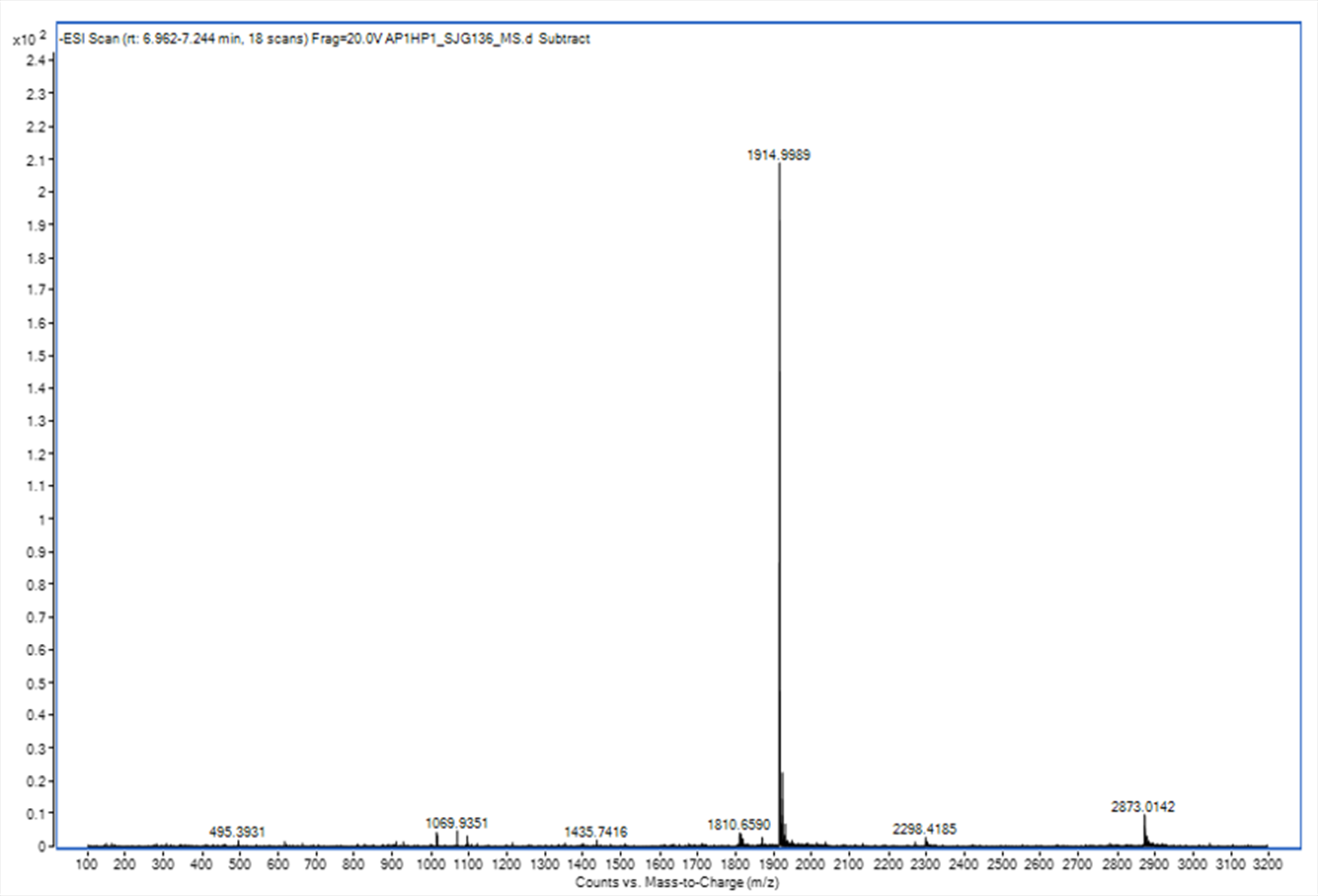

Supplement: S13 Fig — Full ESI mass spectrum for the chromatographic peak eluting at 7.1 min for Hairpin-5 from S8 Fig at collision energy 40. The signal at m/z 1915 corresponds to the triply charged [M-3H]3- ion of Hairpin-5. (TIF) [file pone.0152303.s013.tif]

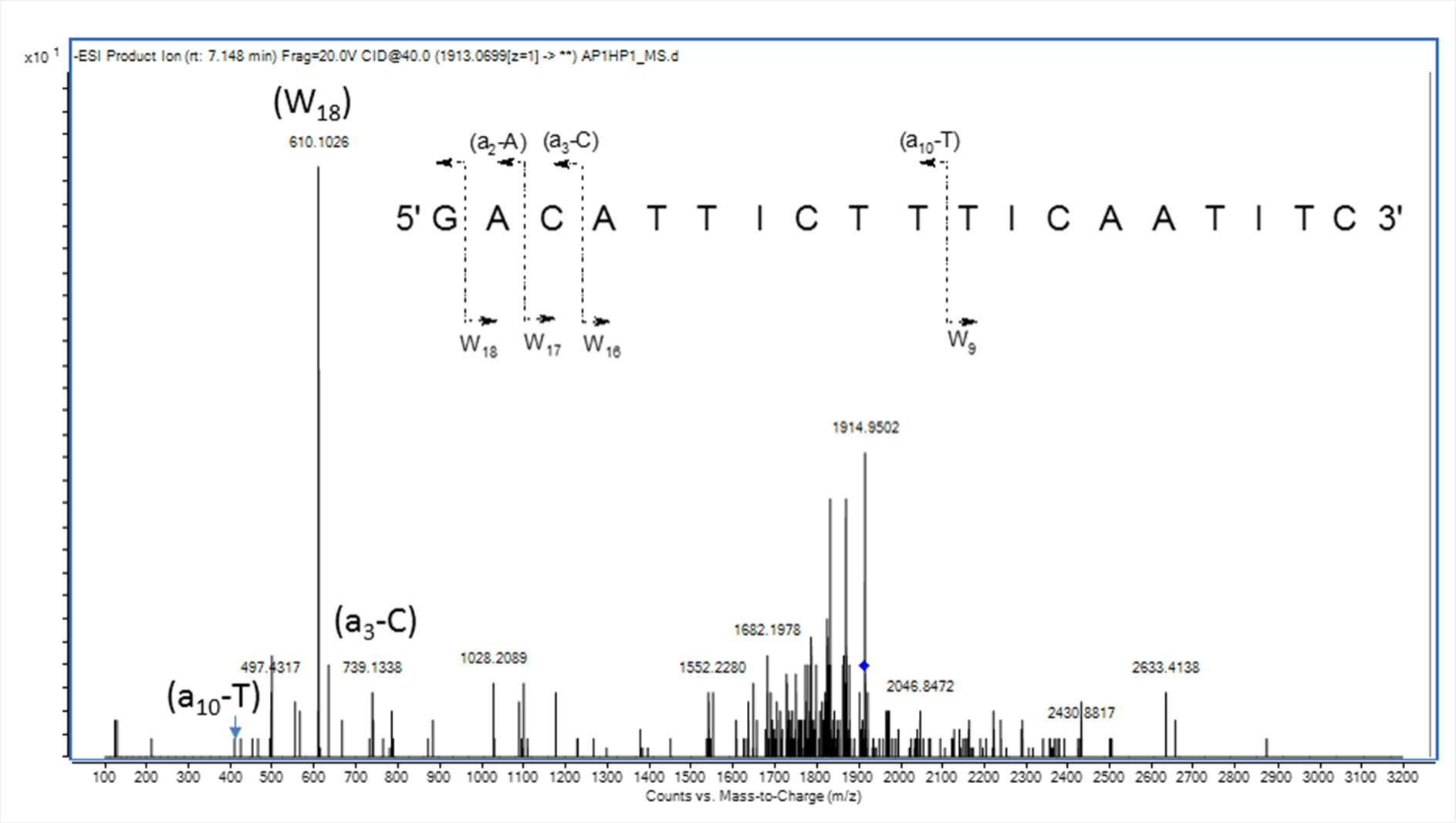

Supplement: S14 Fig — MS/MS spectrum for m/z 1913 corresponding to the chromatographic peak at 7.1 min for Hairpin-5 from S8 Fig at collision energy 40. (TIF) [file pone.0152303.s014.tif]

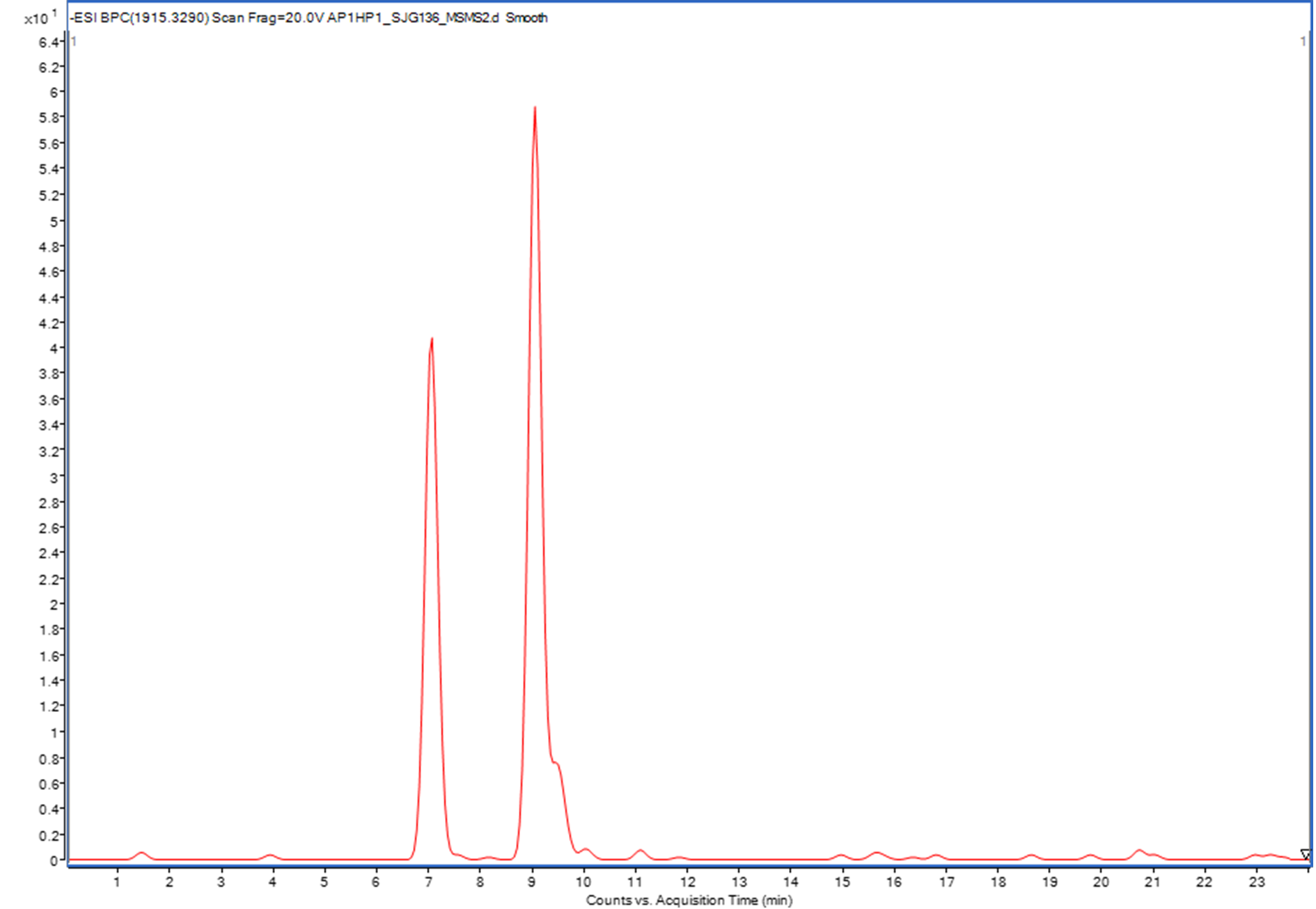

Supplement: S15 Fig — Reconstructed ion chromatogram of m/z 1914 from SJG-136/DNA adduct formation at a molar ratio of 4:1 after 12h incubation, with Hairpin-5 eluting at 7.1 min and the SJG-136/DNA adduct eluting at 9.1 min. (TIF) [file pone.0152303.s015.tif]

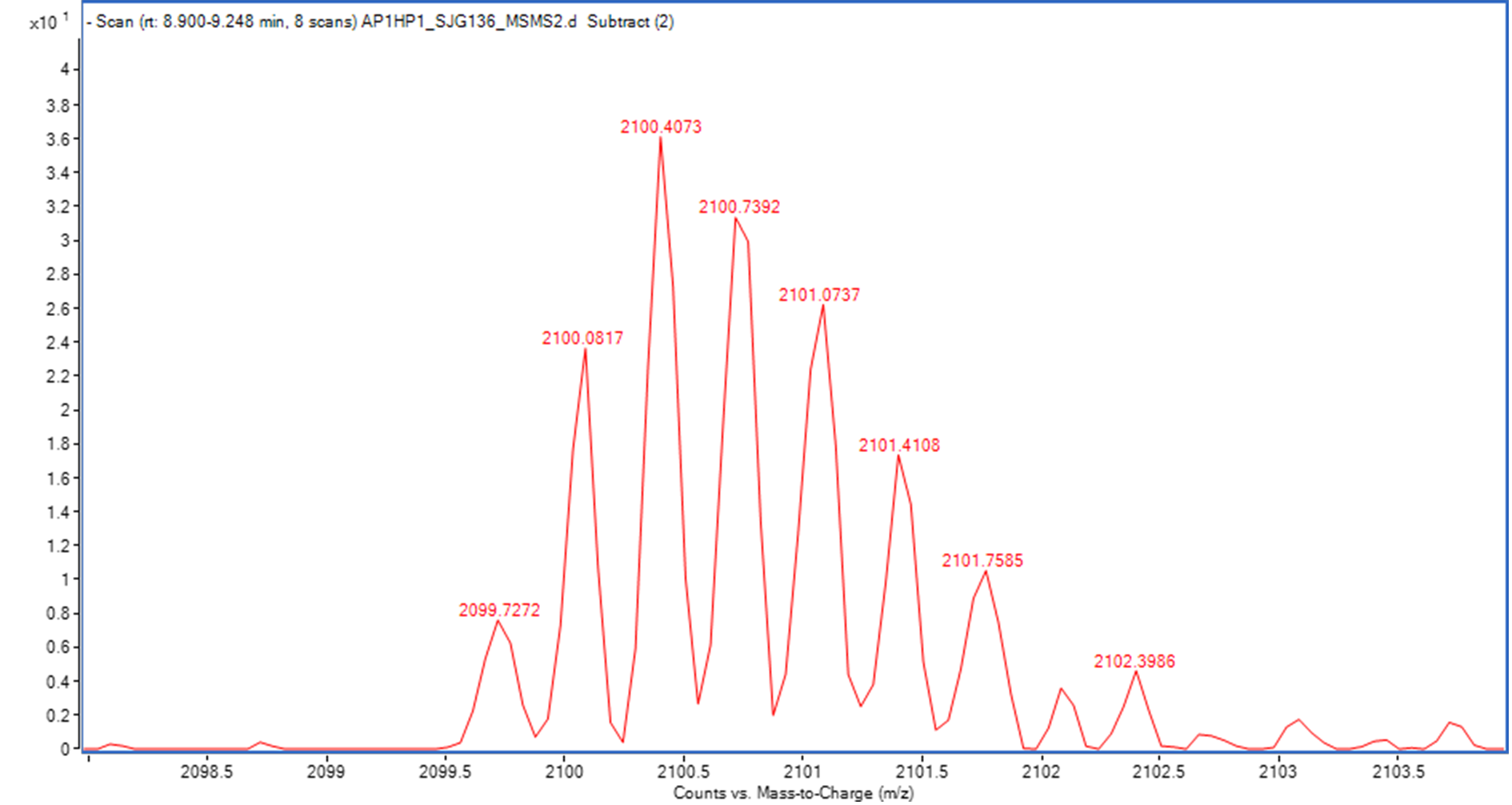

Supplement: S16 Fig — Full ESI mass spectrum for the chromatographic peak at 9.1 min (see S15 Fig) expanded in the mass range from m/z 2090 to 2106. The signal at m/z 2099.72 corresponds to the triply charged [M-3H]3- ion of SJG-136/Hairpin-5. (TIF) [file pone.0152303.s016.tif]

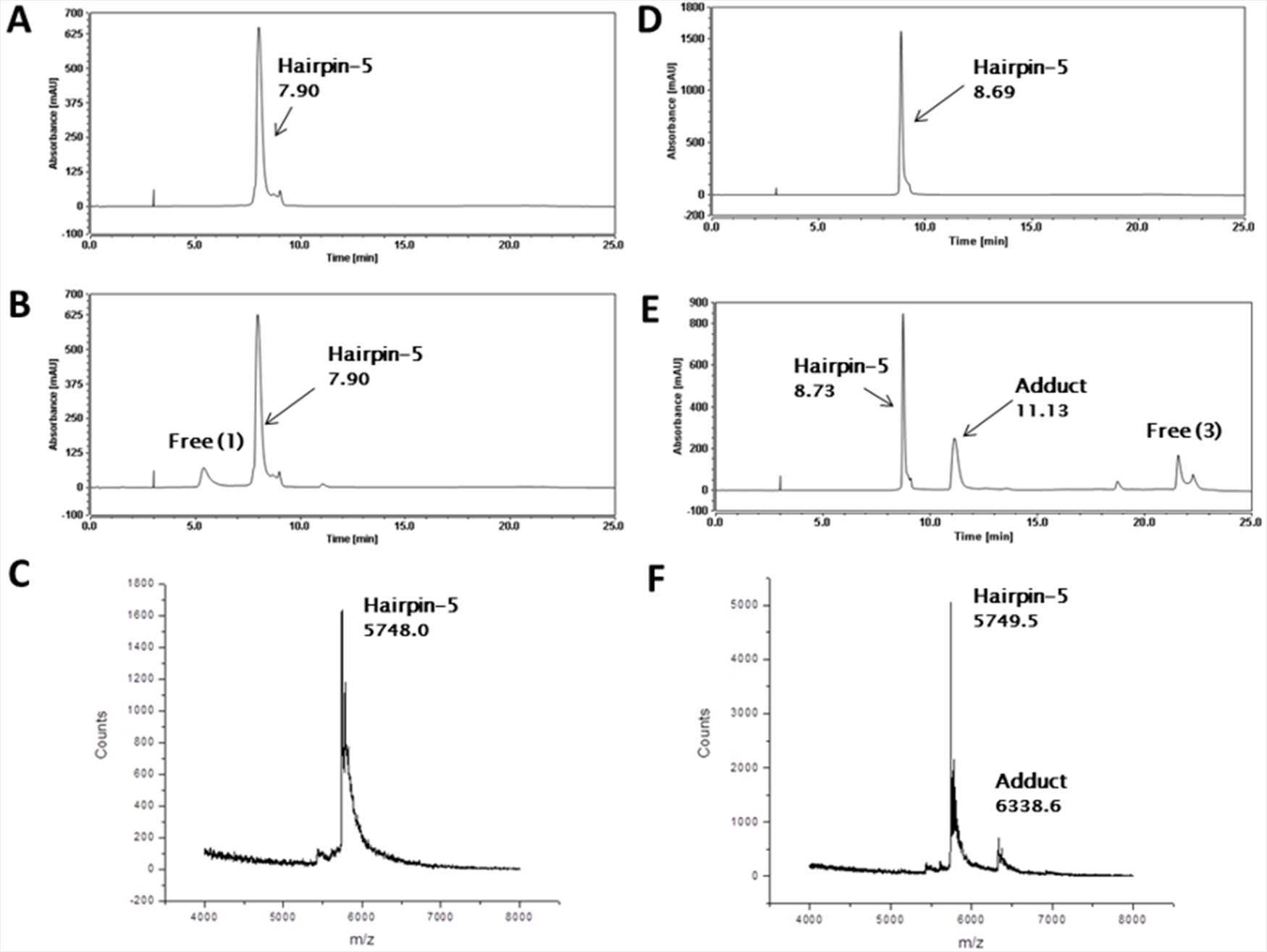

Supplement: S17 Fig — A, HPLC chromatogram showing annealed Hairpin-5 at RT 7.90 min; B, Annealed Hairpin-5 after incubating with 1 for 24 hours showing no adduct formation after 24 hours; C, MALDI-TOF spectrum of 1 with Hairpin-5 confirming that no adduct had formed (Hairpin-5 observed mass: 5748.0 m/z, theoretical mass: 5748.8 m/z); D, Annealed Hairpin-5 at RT 8.69 min; E, Annealed Hairpin-5 after incubating with 3 for 24 hours showing the appearance of an adduct peak at RT 11.13 min with approximately 23% complete reaction after 24 hours; F, MALDI-TOF spectrum of 3/Hairpin-5 confirming the identity of adduct formation (Hairpin-5 observed mass: 5749.5 m/z, theoretical mass: 5748.8 m/z, Hairpin-5 adduct observed mass: 6338.6 m/z, theoretical mass: 6339.4 m/z). The observed differences in the RT of Hairpin-5 alone (i.e., S17A Fig and S17D Fig) occurred due to mobile phase changes. (TIF) [file pone.0152303.s017.tif]

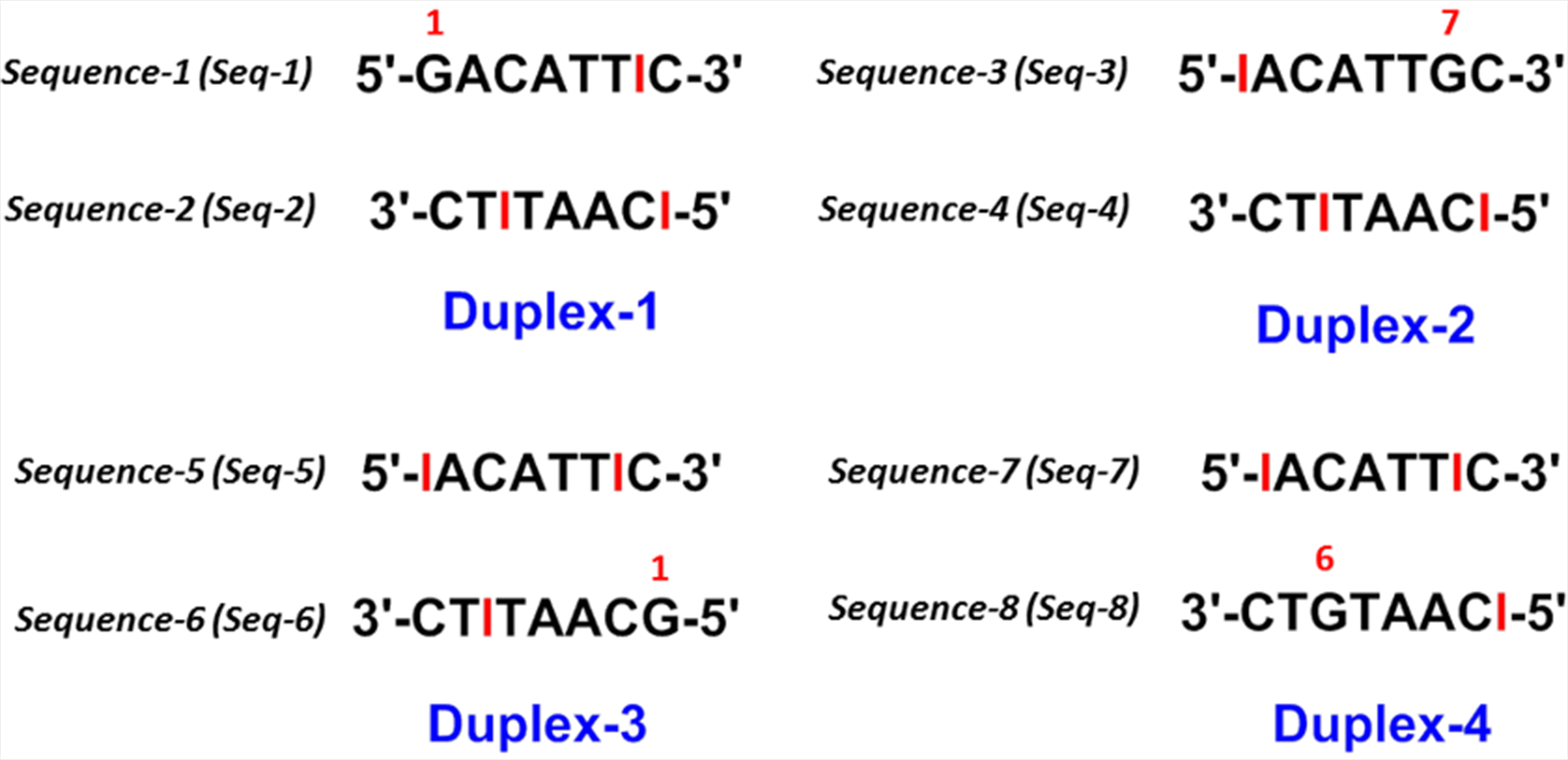

Supplement: S18 Fig — Duplexes 1–4 are analogous to Hairpins 5–8 (Fig 2 of main paper), in that three of the four guanines have been replaced with inosines. The duplexes are 8 base pairs in length. (TIF) [file pone.0152303.s018.tif]

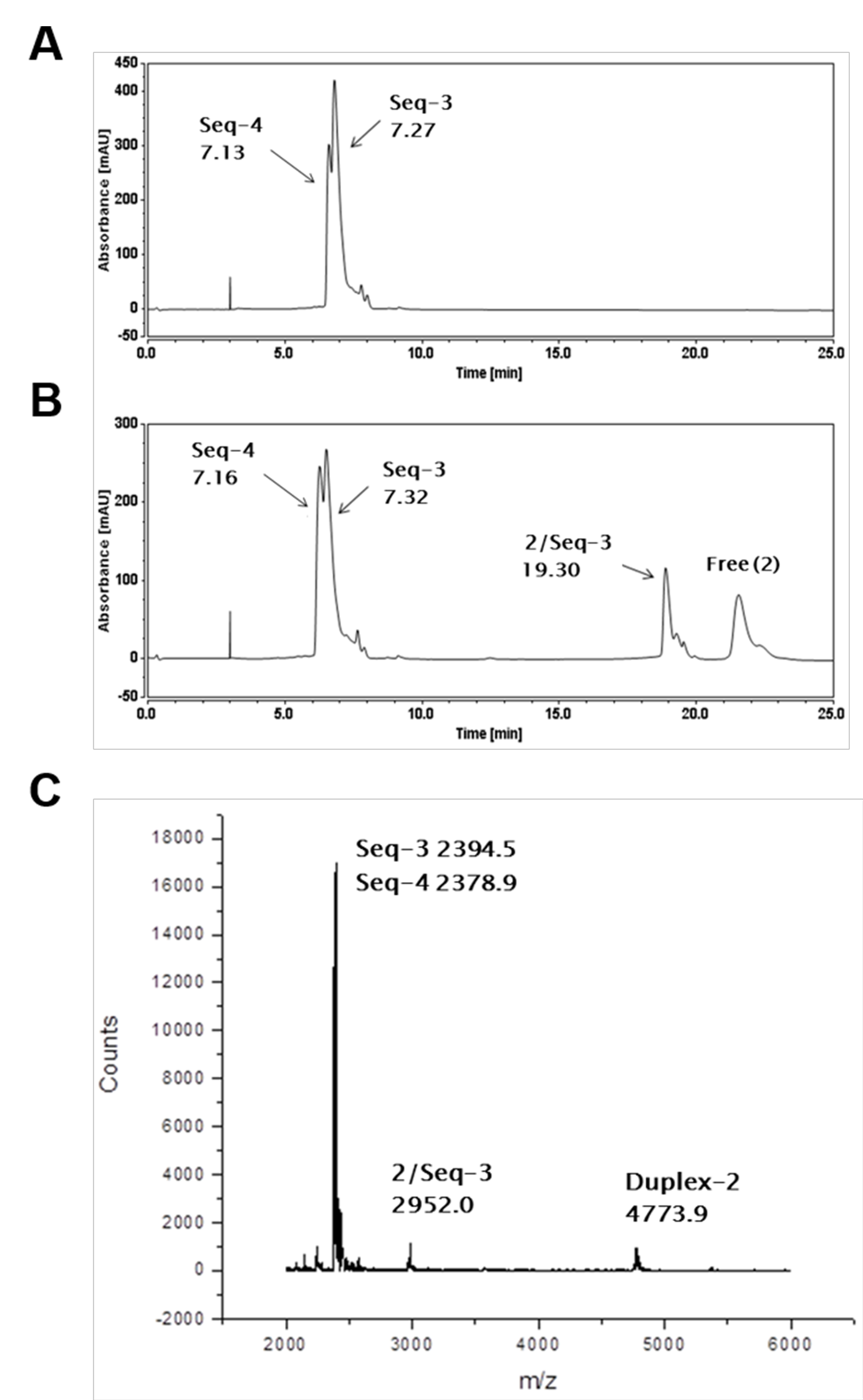

Supplement: S19 Fig — A, HPLC chromatogram of annealed Duplex-2 at RT 7.13 min (Seq-4) and RT 7.27 min (Seq-3); B, Annealed Duplex-2 after incubating with 2 for 24 hours showing a new adduct peak at RT 19.30 min (2/Seq-3), with the reaction not complete after 24 hours; C, MALDI-TOF spectrum of 2 with Duplex-2, confirming the stoichiometry of the adduct formed; single strands of Duplex-2, observed masses: Seq-4, 2378.9 m/z and Seq-3, 2394.5 m/z (theoretical mass: Seq-4 2379.6 m/z and Seq-3 2394.6 m/z), and Duplex-2 observed mass: 4773.9 m/z (theoretical mass: 4774.2 m/z), 2/Seq-3 adduct observed mass: 2952.0 m/z (theoretical mass: 2951.21 m/z). (TIF) [file pone.0152303.s019.tif]

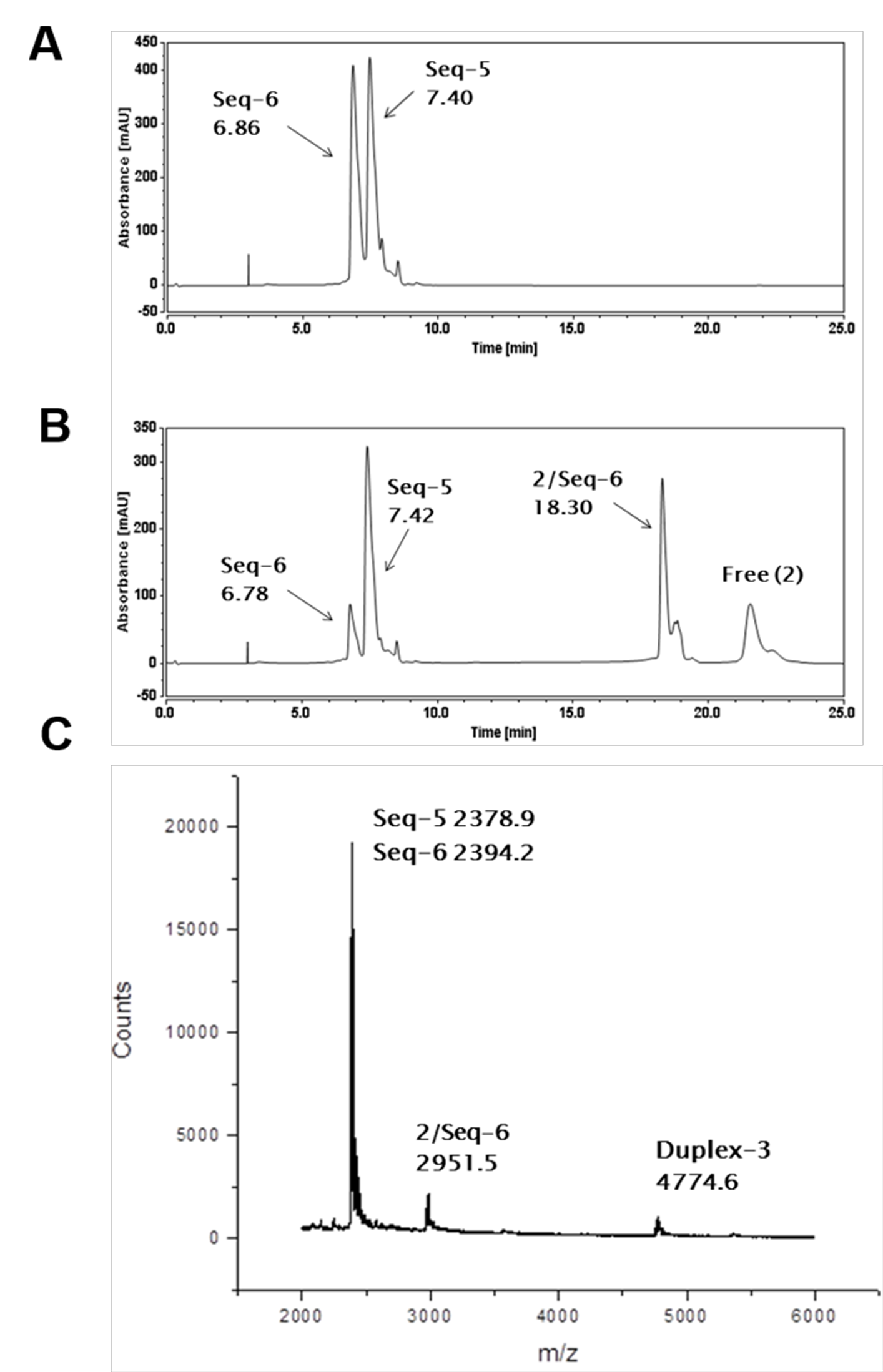

Supplement: S20 Fig — A, HPLC chromatogram of annealed Duplex-3 at RT 6.86 min (Seq-6) and RT 7.40 min (Seq-5); B, Annealed Duplex-3 after incubating with 2 for 24 hours showing a new adduct peak at RT 18.30 min (2/Seq-6), with the reaction not complete after 24 hours; C, MALDI-TOF spectrum of 2 with Duplex-3, confirming the stoichiometry of the adduct formed; single strands of Duplex-3, observed masses: Seq-5, 2378.9 m/z and Seq-6, 2394.2 m/z (theoretical mass: Seq-5 2379.6 m/z and Seq-6 2394.6 m/z), and Duplex-3 observed mass: 4774.6 m/z (theoretical mass: 4774.2 m/z), 2/Seq-6 adduct observed mass: 2951.5 m/z (theoretical mass: 2951.21 m/z). (TIF) [file pone.0152303.s020.tif]

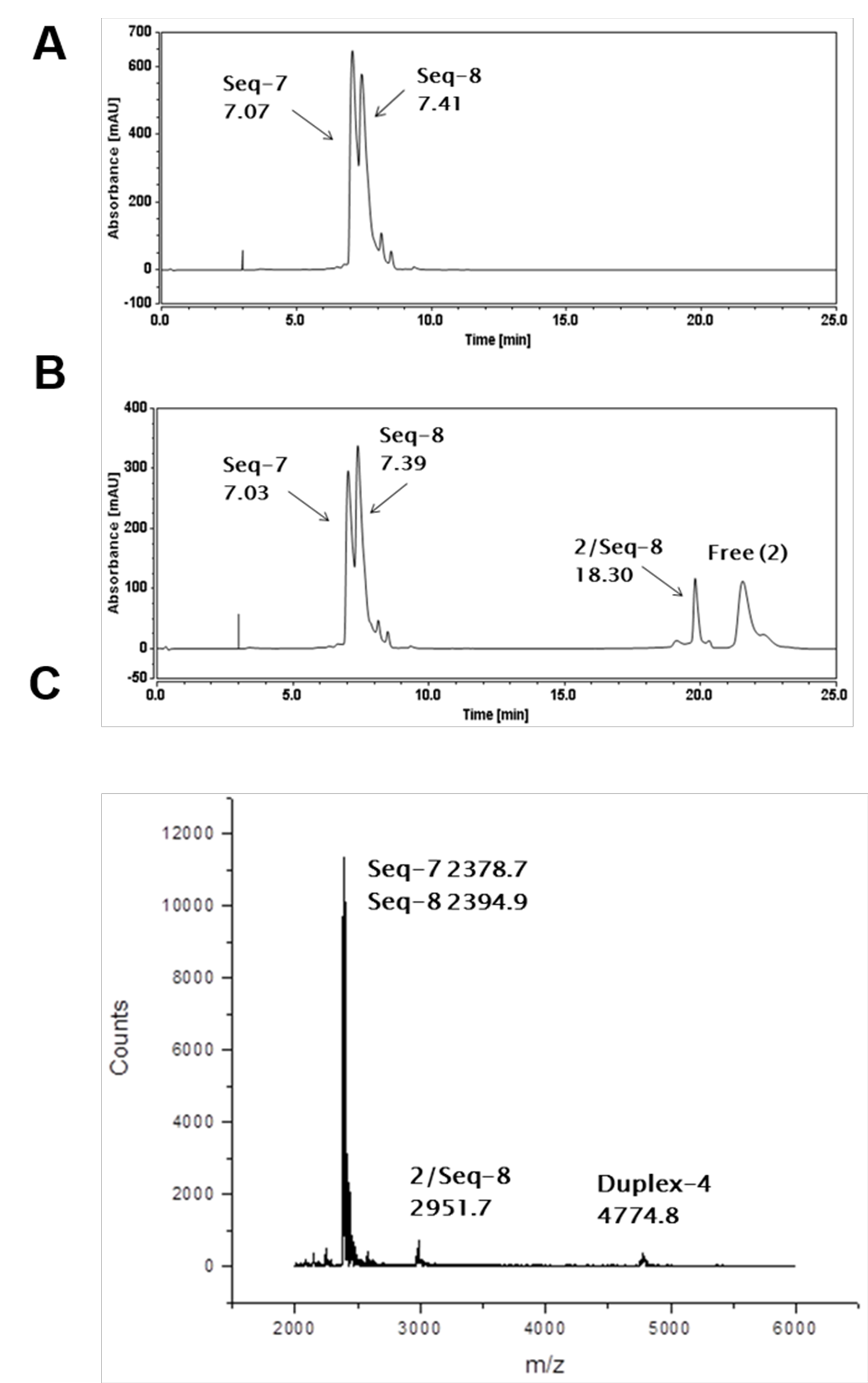

Supplement: S21 Fig — A, HPLC chromatogram of annealed Duplex-4 at RT 7.07 min (Seq-8) and RT 7.4 min (Seq-7); B, Annealed Duplex-4 after incubating with 2 for 24 hours showing a new adduct peak at RT 18.30 min (2/Seq-8), with the reaction not complete after 24 hours; C, MALDI-TOF spectrum of 2 with Duplex-4, confirming the stoichiometry of the adduct formed; single strands of Duplex-4, observed masses: Seq-7, 2378.7 m/z and Seq-8, 2394.9 m/z (theoretical mass: Seq-7 2379.6 m/z and Seq-8 2394.6 m/z), and Duplex-4 observed mass: 4774.8 m/z (theoretical mass: 4774.2 m/z), 2/Seq-8 adduct observed mass: 2951.7 m/z (theoretical mass: 2951.21 m/z). (TIF) [file pone.0152303.s021.tif]

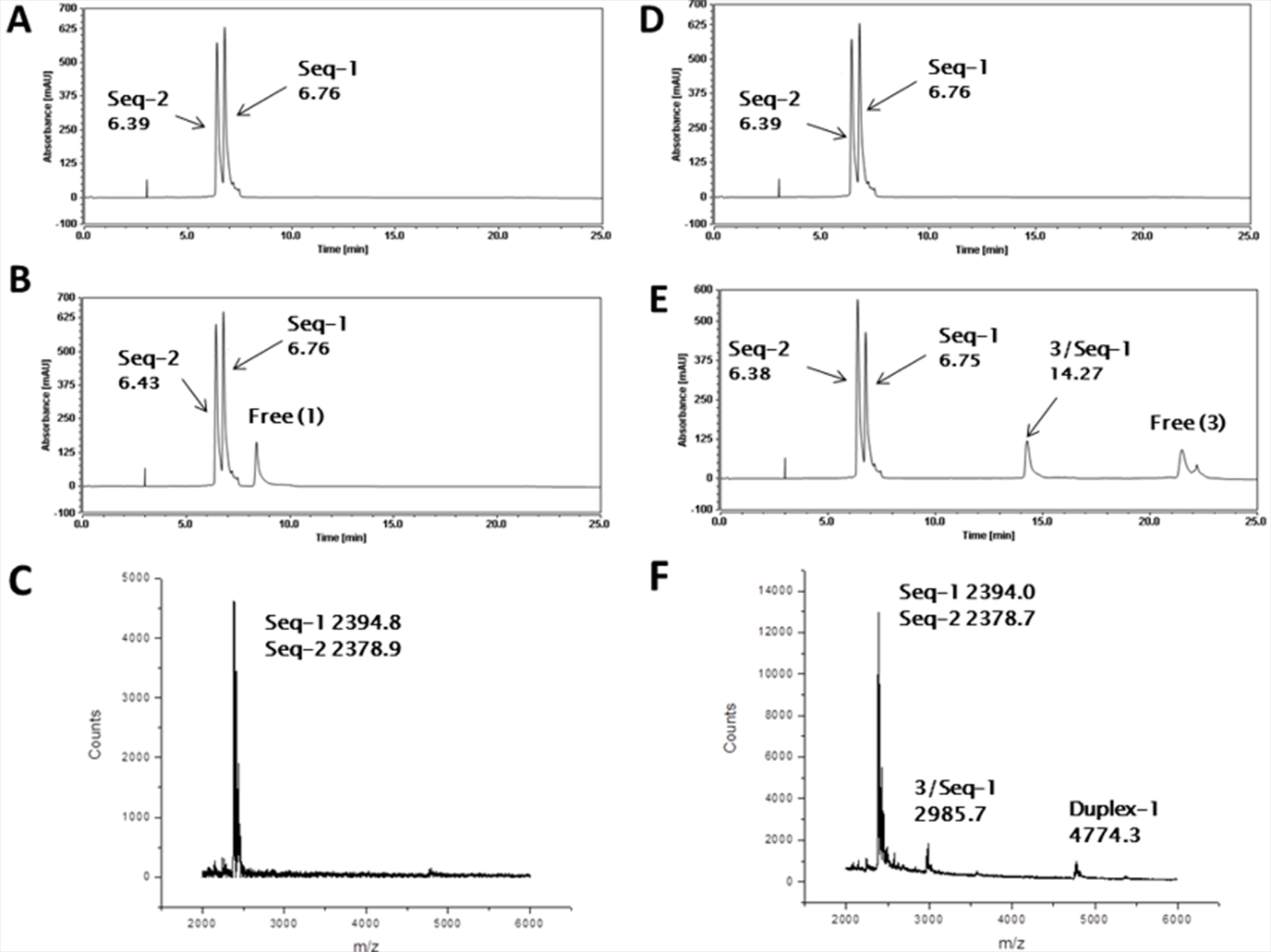

Supplement: S22 Fig — A, HPLC chromatogram of annealed Duplex-1 at RT 6.39 min (Seq-2) and RT 6.76 min (Seq-1); B, After incubation with 1 for 24 hours showing no reaction; C, MALDI-TOF spectrum of 1 with Duplex-1 confirming no reaction; Single strands of Duplex-1 observed masses: Seq-2, 2378.9 m/z and Seq-1, 2394.8 m/z (theoretical masses: Seq-2, 2379.6 m/z and Seq-1, 2394.6 m/z); D, Annealed Duplex-1 at RT 6.39 min (Seq-2) and 6.76 min (Seq-1); E, Annealed Duplex-1 after incubating with 3 for 24 hours showing a new peak at RT 14.27 min; F, MALDI-TOF spectrum of 3 with Duplex-1 confirming the stoichiometry as the 1:1 3/Seq-1 adduct; Single strands of Duplex-1 observed masses: Seq-2, 2378.7 m/z and Seq-1, 2394.0 m/z (theoretical masses: Seq-2, 2379.6 m/z and Seq-1, 2394.6 m/z) and double-stranded Duplex-1 observed mass of 4774.3 m/z (theoretical mass: 4774.2 m/z), 3/Seq-1 adduct observed mass of 2985.7 m/z (theoretical mass: 2985.2 m/z). (TIF) [file pone.0152303.s022.tif]

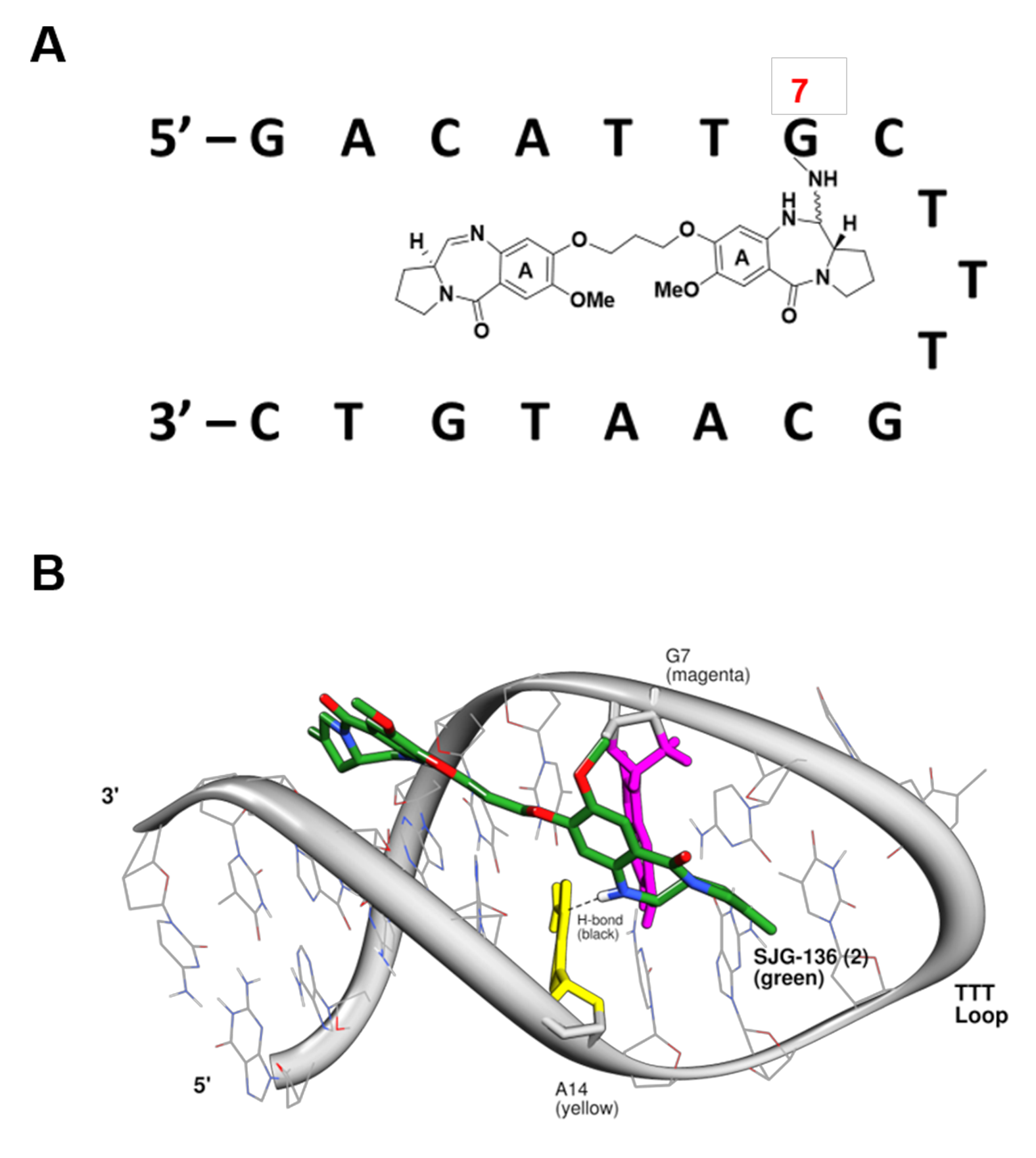

Supplement: S23 Fig — Schematic and low energy snapshot of a molecular dynamics simulation showing the accommodation of 2 (green sticks) in the minor groove while covalently bonded to G7 of Hairpin-1 (see Fig 2 of main paper). The NH group of the PBD formed a H-bond with the adjacent A14 adenine base (yellow), and the molecule maintained good isohelicity with the minor groove for the duration of the simulation (10 ns). (TIF) [file pone.0152303.s023.tif]

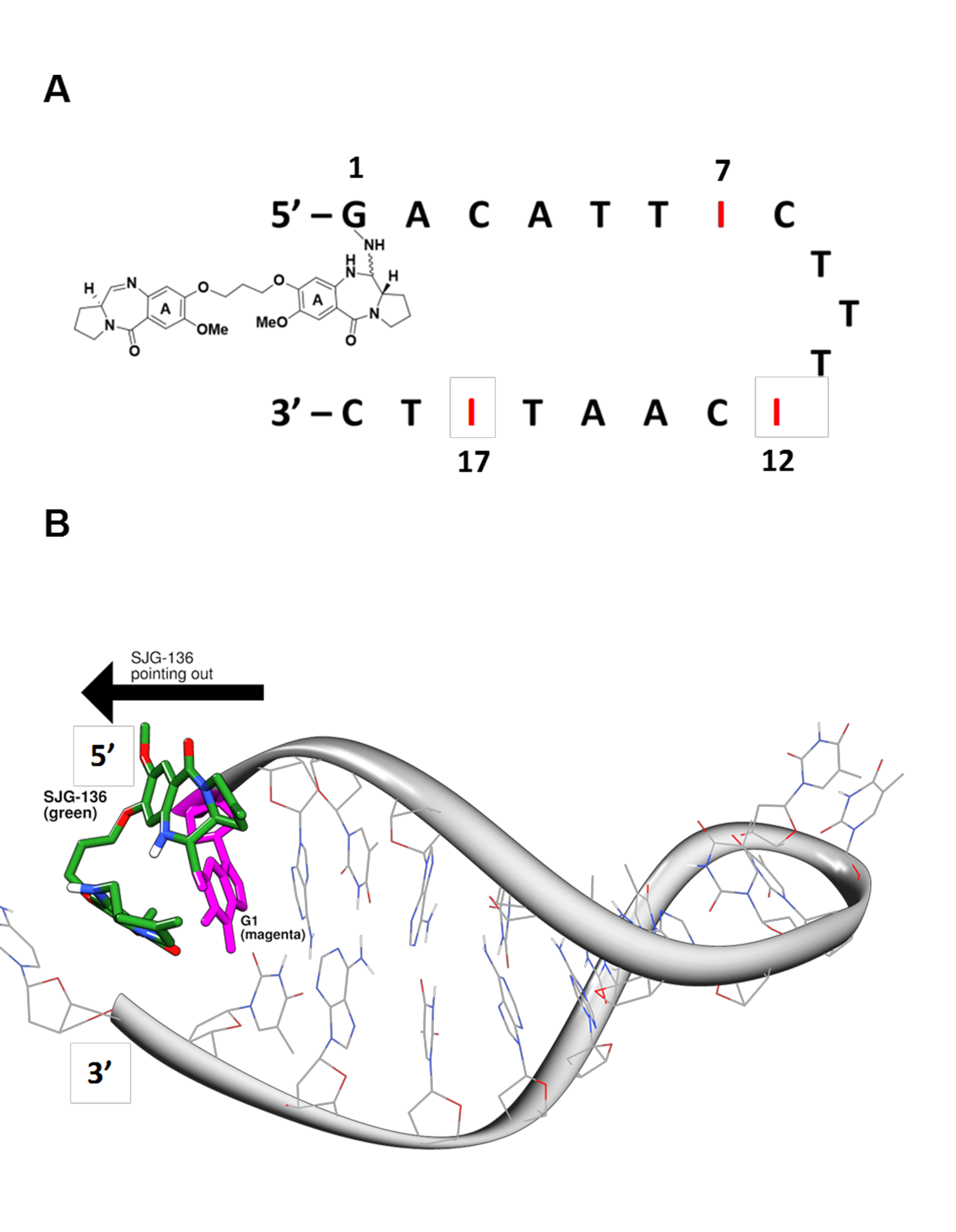

Supplement: S24 Fig — A, Schematic model of 2 (green) covalently bound to G1 (magenta) of Hairpin-5 with the A-ring pointing away from the TTT-loop (A-ring-3’ orientation), and the second PBD of the dimer orienting outside of the minor groove; B, Low energy snapshot of 10 ns molecular dynamics simulation illustrating the second PBD of 2 orienting outside of the DNA minor groove. (TIF) [file pone.0152303.s024.tif]

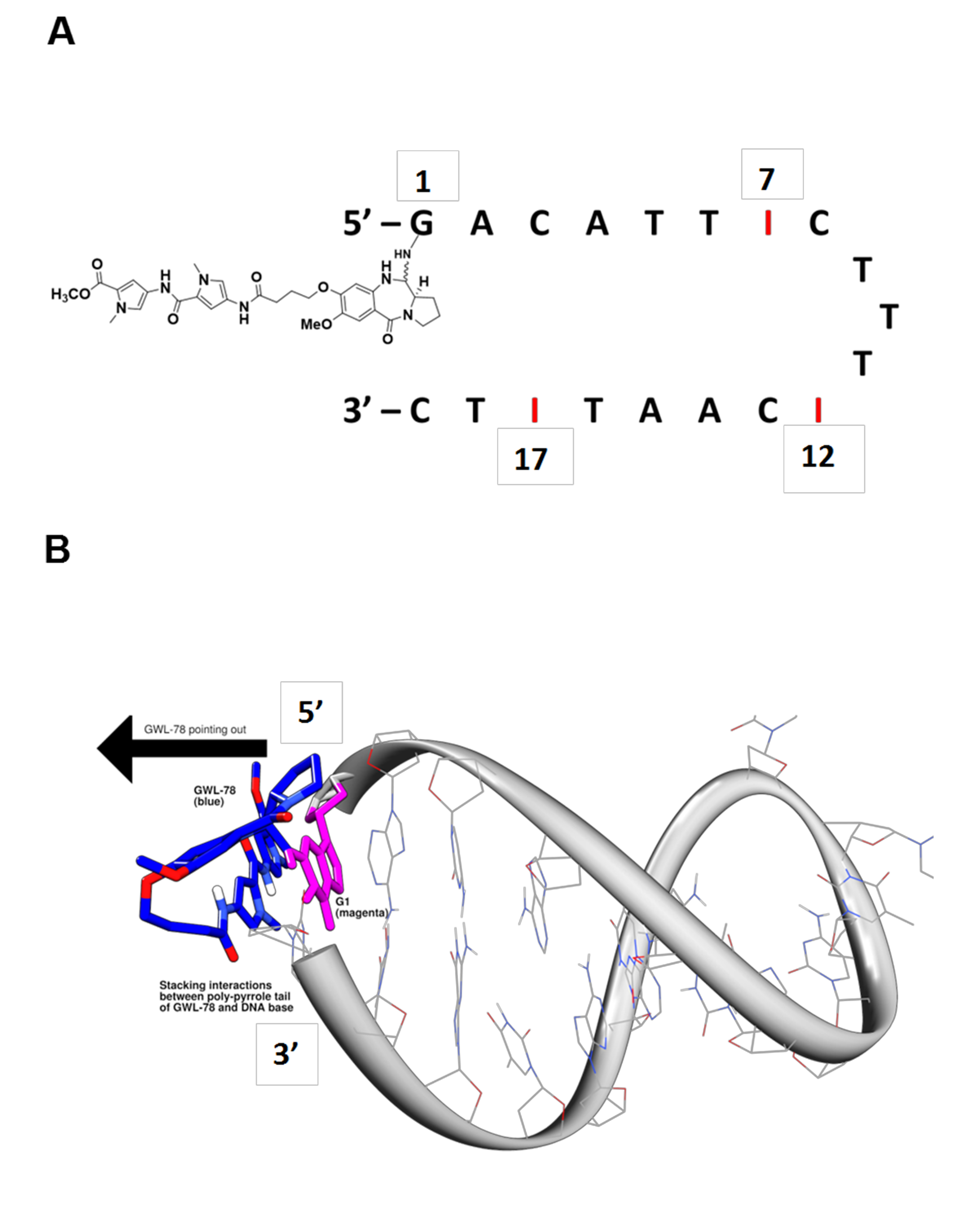

Supplement: S25 Fig — A, Schematic model of 3 (blue) covalently bound to G1 (magenta) of Hairpin-5 with the A-ring pointing away from the TTT-loop (A-ring-3’ orientation), and the C8-poly-pyrrole chain orienting outside of the minor groove; B, Low energy snapshot of a 10 ns molecular dynamics simulation illustrating the C8-poly-pyrrole chain of 3 orienting outside of the DNA minor groove and forming stacking interactions with the G1 base. (TIF) [file pone.0152303.s025.tif]

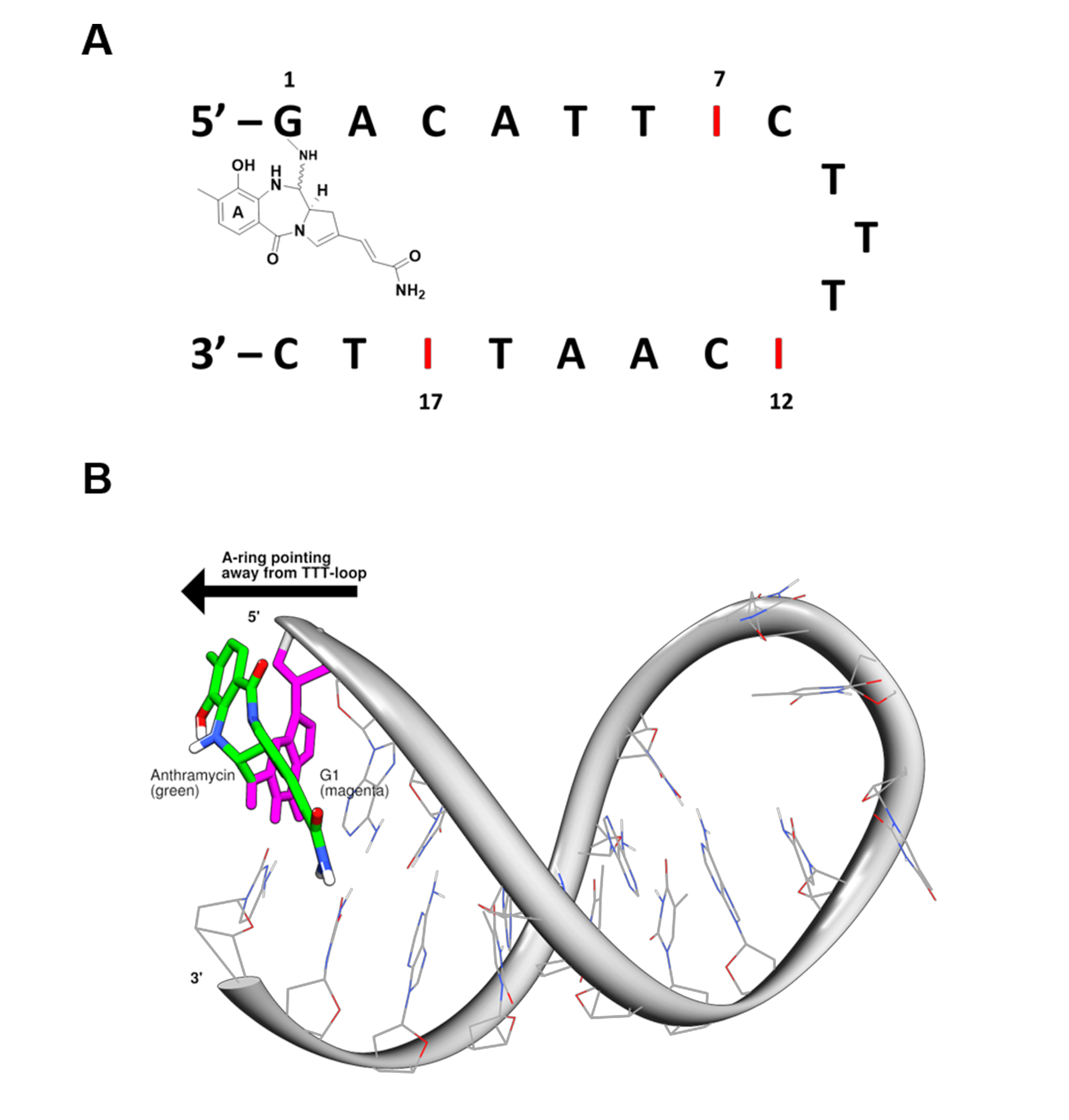

Supplement: S26 Fig — A, Schematic model of 1 (light green) covalently bound to G1 (magenta) of Hairpin-5 with the A-ring pointing away from the TTT-loop (A-ring-3’ orientation); B, Low energy snapshot of a 10 ns molecular dynamics simulation illustrating anthramycin (1) potentially bound to the G1 base through interactions with a doublet rather than a traditional triplet. (TIF) [file pone.0152303.s026.tif]

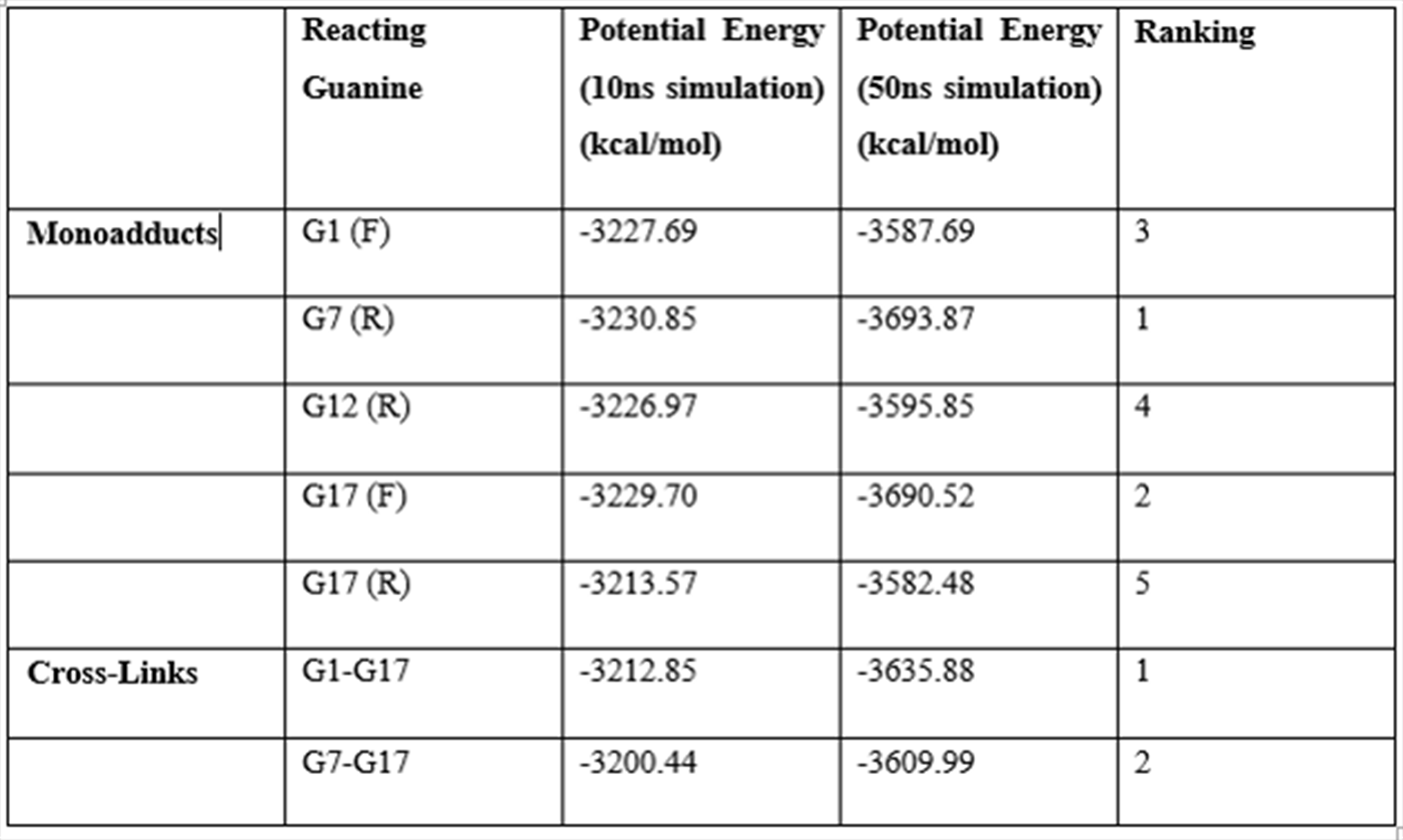

Supplement: S27 Fig — Calculations were undertaken with SJG-136 (2) covalently bound to every potential reacting guanine in the AP-1 consensus sequence. The order of preference of binding was identical in both 10ns and 50ns simulations. (TIF) [file pone.0152303.s027.tif]
